# Analysis of *Anasplatyrhynchos* genome resequencing data reveals genetic signatures of artificial selection

**DOI:** 10.1371/journal.pone.0211908

**Published:** 2019-02-08

**Authors:** Tieshan Xu, Lihong Gu, Haopeng Yu, Xuefei Jiang, Yunsheng Zhang, Xiaohui Zhang, Guang Rong, Zhengkui Zhou, Kyle M. Schachtschneider, Shuisheng Hou

**Affiliations:** 1 Tropical Crop Genetic Resource Research Institute, Chinese Academy of Tropical Agricultural Sciences, Danzhou, P.R. China; 2 Institute of Animal Science, Chinese Academy of Agricultural Sciences, Beijing, P.R. China; 3 Institute of Animal Science & Veterinary, Hainan Academy of Agricultural Science, Haikou, P.R. China; 4 West China Biomedical Big Data Center, West China Hospital/West China School of Medicine, Sichuan University, Chengdu, P.R. China; 5 Institute of Tropical Agriculture and Forestry, Hainan University, Haikou, P.R. China; 6 College of Animal Science, Henan University of Science and Technology, Luoyang P.R. China; 7 Department of Radiology, University of Illinois at Chicago, Chicago, Illinois, United States of America; Northwest A&F University, CHINA

## Abstract

Three artificially selected duck populations (AS), higher lean meat ratios (LTPD), higher fat ratios (FTPD) and higher quality meat (CMD), have been developed in China, providing excellent populations for investigation of artificial selection effects. However, the genetic signatures of artificial selection are unclear. In this study, we sequenced the genome sequences of these three artificially selected populations and their ancestral population (mallard, M). We then compared the genome sequences between AS and M and between LTPD and FTPD using integrated strategies such as anchoring scaffolds to pseudo-chromosomes, mutation detection, selective screening, GO analysis, qRT-PCR, and protein multiple sequences alignment to uncover genetic signatures of selection. We anchored duck scaffolds to pseudo-chromosomes and obtained 28 pseudo-chromosomes, accounting for 84% of duck genome in length. Totally 78 and 99 genes were found to be under selection between AS and M and between LTPD and FTPD. Genes under selection between AS and M mainly involved in pigmentation and heart rates, while genes under selection between LTPD and FTPD involved in muscle development and fat deposition. A heart rate regulator (*HCN1*), the strongest selected gene between AS and M, harbored a GC deletion in AS and displayed higher mRNA expression level in M than in AS. *IGF2R*, a regulator of skeletal muscle mass, was found to be under selection between FTPD and LTPD. We also found two nonsynonymous substitutions in *IGF2R*, which might lead to higher *IGF2R* mRNA expression level in FTPD than LTPD, indicating the two nonsynonymous substitutions might play a key role for the regulation of duck skeletal muscle mass. Taken together, these results of this study provide valuable insight for the genetic basis of duck artificial selection.

## Introduction

Based on Fisher’s theory of natural selection in 1930, traits are associated with evolutionary fitness, such as morphology and complex physiology [1]. Validation of Fisher’s theorem has been demonstrated by the successful production of improved breeds for a wide range of species using artificial selection, including pigs [2], chickens [3], cattle [4], sheep [5], and inbred rat strains [6]. Currently, it has been shown that next generation sequencing technologies are very effective in identifying the genetic basis of improved or domesticated species. Next generation sequencing technologies are therefore widely used to explore genetic basis in a number of species including passenger pigeons [7], pigs [8], chickens [9], dogs [10], rabbits [11], polar bears [12] and cormorants [13].

Ducks (*Anasplatyrhynchos*) dissociated from chickens, zebra finches, and turkeys approximately 90–100 million years ago [14]. Ducks display great differences in morphology [15], physiology [16], and behavior [17] comparing to chickens, zebra finches, and turkeys due to a long period of differentiation selection. In addition, remarkable changes induced by natural and/or artificial selection have occurred in domesticated duck breeds compared to their wild ancestor (mallard) [18]. In 2013, the duck genome was sequenced [19] (http://www.ensembl.org/Anas_platyrhynchos/Info/Index), providing a platform for investigation of the mechanisms underlying artificial selection and domestication in ducks. It greatly facilitates us to investigate the genetic basis underlying differential traits through comparison of genome sequences of different duck populations.

China is the No. 1 country in the world in terms of the production and consumption of ducks, with the total number of ducks produced in China accounting for more than 90% of global duck production in 2014 (2,121,194,000 in China out of 2,324,224,000 worldwide) (FAO, http://www.fao.org/faostat/en/#data/QL). Pekin duck, a typical variety in China, was first bred in the 1960s by the Chinese Academy of Agricultural Sciences. Since then two new strains, lean-type Pekin ducks (LTPD) and fat-type Pekin ducks (FTPD), have been developed [20]. They differ significantly in lean meat ratio and fatness ratio (S1 Table). In addition, using the Sheldrake breed as a breeding material, a new duck strain named the China Micro-duck (CMD) characterized with high quality meat and white feather has also been developed in China.

To investigate the genetic signatures underlying artificial selection in these new duck breeds, genomic sequences of all three breeds (LTPD, FTPD, and CMD, referred to collectively as the artificial selection population (AS)) were compared with their ancestor (mallard, M). These populations display many differential characteristics and phenotypes (S1 Table). For example, LTPD and FTPD populations differ significantly in skeletal muscle development and fat deposition, whereas AS and M populations differ in feather color and heart rates. Therefore, we compared the genomic sequences of LTPD and FTPD to investigate the genetic signatures underlying differential skeletal muscle development and fat deposition, and the genomic sequences of AS and M to investigate the genetic signatures controlling feather color and heart rates.

In this study, we carried out whole genome resequencing for the four duck populations using pools of genomic DNA with an average coverage of ~ 40× per pool. SNPs were detected for each population. Genomic regions under selection and genes overlapping these regions were identified and the functional effects of mutations harbored by selected genes were also investigated. We identified a two base (GC) deletion in *HCN1* likely responsible for decreased heart rates observed in AS compared to M population. In addition, two SNPs harbored by *IGF2R* may contribute to increased skeletal muscle percentage. These results provide a better understanding of the mechanism underlying the altered skeletal muscle development, fat deposition, feather colors, and heart rates during artificial selection in ducks.

## Results and discussion

### Anchoring duck scaffolds to pseudo-chromosomes

**Fig 1:**
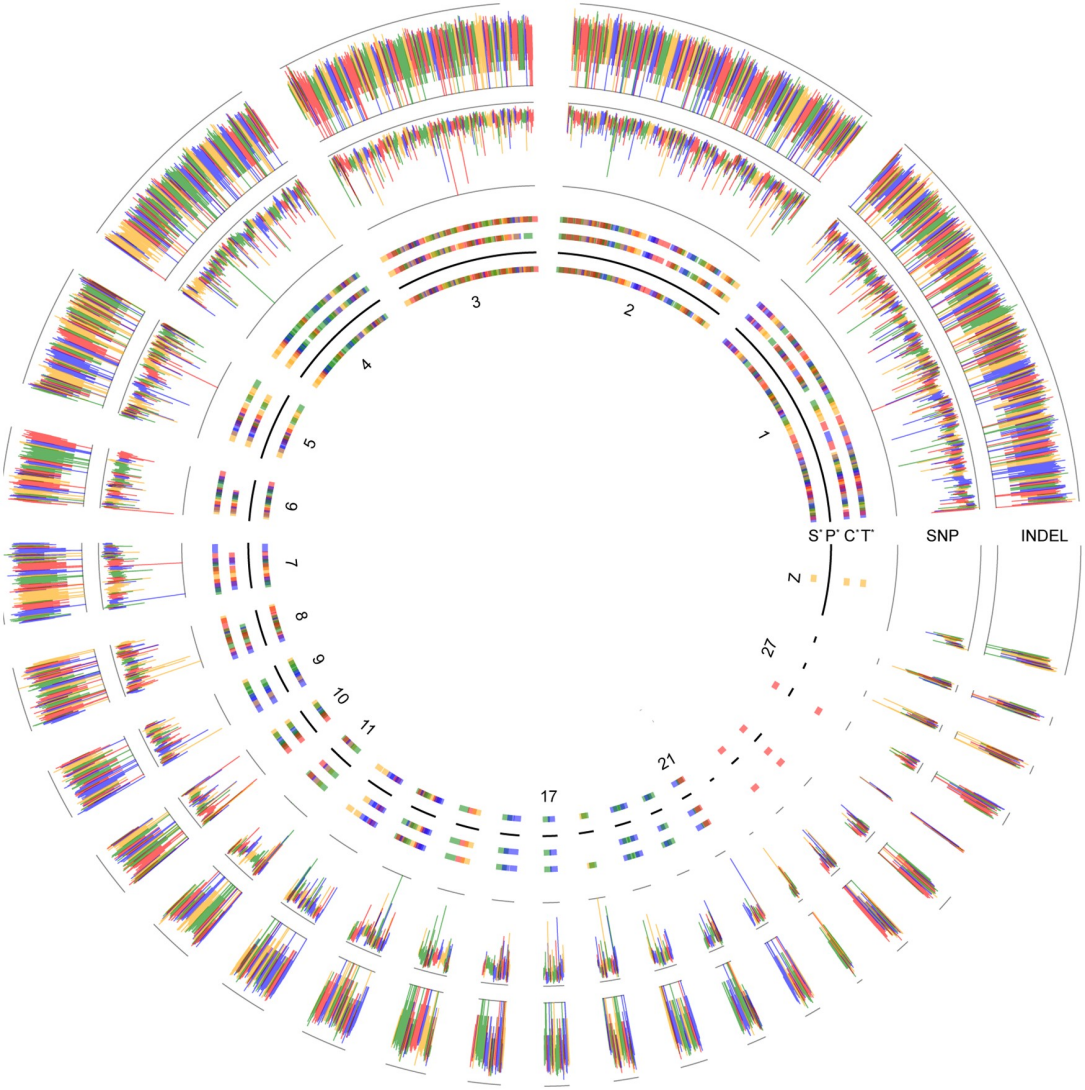
The anchoring of duck scaffolds to pseudo-chromosomes and the distribution of genomic variation. The inner four circles illustrate the collinearity of Peking duck between chicken and turkey, the anchored scaffolds were shown with different colors. The innermost circle S* shows the anchored scaffolds ordered by pseudo-chromosomes. Circle P* represents the anchored duck pseudo-chromosomes. Circle C* and T* represent the distribution of corresponding scaffolds from the duck genome to chicken and to turkey genomes respectively. **Circle SNP** presents the distribution of SNP frequency within a bin size of 1k along each pseudo-chromosome. **Circle INDEL** presents the distribution of INDEL frequency within a bin size of 1k along each pseudo-chromosome.

The current duck genome (http://www.ensembl.org/Anas_platyrhynchos/Info/Index) lacks the linkage information to align scaffolds at the chromosome level, which impedes the identification of continuous genomic signatures under selection and genes overlapping these signatures. Comparative genomics analysis has revealed that ducks have the closest genetic relationship with chickens and turkeys, whose genomes have been assembled with assigned chromosomes [21]. Based on chromosomal collinearity between duck and chicken, and between duck and turkey, we assigned duck scaffolds to pseudo-chromosomes. We obtained 28 pseudo-chromosomes representing 27 autosomes and one sex chromosome (chromosome Z) (Fig 1, S2 Table), which accounts for 70% (28 out of 40) of the duck chromosomes [22]. The 28 pseudo-chromosomes spanned ~923 Mb, accounting for 84% of the assembled duck genome (1,104 Mb). The remaining scaffolds (length < 2 kb and not assigned to pseudo-chromosomes) were randomly linked as pseudo-chromosome UN and used for downstream analysis.

### A load of variants were detected in duck genome

We performed Pool-seq to study the genomic variation of the four duck populations (LTPD, FTPD, CMD, and M; n = 30/group). The DNA pools for each population were paired-end sequenced to generate an average of ~40x coverage per pool, resulting in a total coverage of 168.44x (185.96 Gb; S3 and S4 Tables). Mapping the reads to the duck genome resulted in an average alignment rate of 89.71%, of which 86.75% were uniquely mapped (Table 1).

**Table 1:** Alignment statistics.

Samples	Read pair count	Mapped reads		Uniquely mapped reads		Multiply mapped reads	
		count	%	count	%	count	%
FTPD	219,905,659	397,122,739	90.29	191,971,689	87.3	6,316,533	1.44
LTPD	228,789,774	409,502,755	89.49	198,455,153	86.74	6,127,696	1.34
CMD	224,920,852	404,194,537	89.85	195,367,187	86.86	6,339,301	1.41
M	226,178,409	403,597,540	89.22	194,761,536	86.11	6,733,997	1.49
Average	224,948,674	403,604,393	89.71	195,138,891	86.75	6,379,382	1.42

Analysis of genomic mutations, such as SNPs and short insertions and deletions (INDELs), is the basis of investigating the genetic mechanism underlying artificial selection at the genomic level. In this study, we detected SNPs and INDELs located in each of anchored pseudo-chromosomes and pseudo-chromosome UN, and summarized the results in a circular ideogram layout (Fig 1). In total, 11,393,231 high quality SNPs were identified across the four duck populations (Table 2). Of the SNPs harbored by genes (4,188,685), ~95% (3,977,357) were located in introns (S5 Table). In total, 352 nonsense mutations resulting in incomplete and usually nonfunctional proteins were identified, with an additional 1,431 mutations predicted to effect protein functionality (S5 Table). In addition, we identified 620,677 INDELs across the four duck populations (Table 2). Similar to SNPs, the majority of INDELs in gene regions (215,606) were located in intronic regions (S6 Table) with only 0.29% (2,194) of INDELs predicted to highly affect protein functionality (S6 Table). These results indicate only a small proportion of the identified mutations result in altered or loss of gene function and that loss of gene function is not the predominate cause of the differential phenotypes resulting from artificial selection in duck populations, which is consistent with previous reports in rabbits [11], pigs [23] and chickens [9].

**Table 2:** Detected variants for each duck population.

Items		FTPD	LTDP	CMD	M	Total
Assembly coverage (%)		98.71	98.61	98.69	98.61	99.00
Heterozygosity		0.3721	0.3789	0.3816	0.3626	0.3264
SNP	Non-ref alleles	8,011,524	7,791,602	8,293,270	10,324,867	11,393,231
	Unique alleles	13,866	12,934	47,964	165,388	NA
INDELs	Non-ref alleles	475,310	463,809	485,661	569,653	620,677
	Unique alleles	6,570	6,912	9,058	81,150	NA

A phylogenetic tree is a branching diagram or “tree” showing the inferred evolutionary relationships among various biological species or subspecies based upon similarities and differences in genetic characteristics. In this study, we constructed a phylogenetic tree to illustrate the genetic relationships of the four duck populations using SNPhylo software with the default parameters and the allele frequencies of SNPs detected in each population [24] (S1 Fig). The results indicated the LTPD and FTDP populations are the closest genetically related populations, with the CMD population more closely related to the LTPD and FTDP populations than the ancestral M population.

### Many genomic regions associated with artificial selection were found

**Fig 2:**
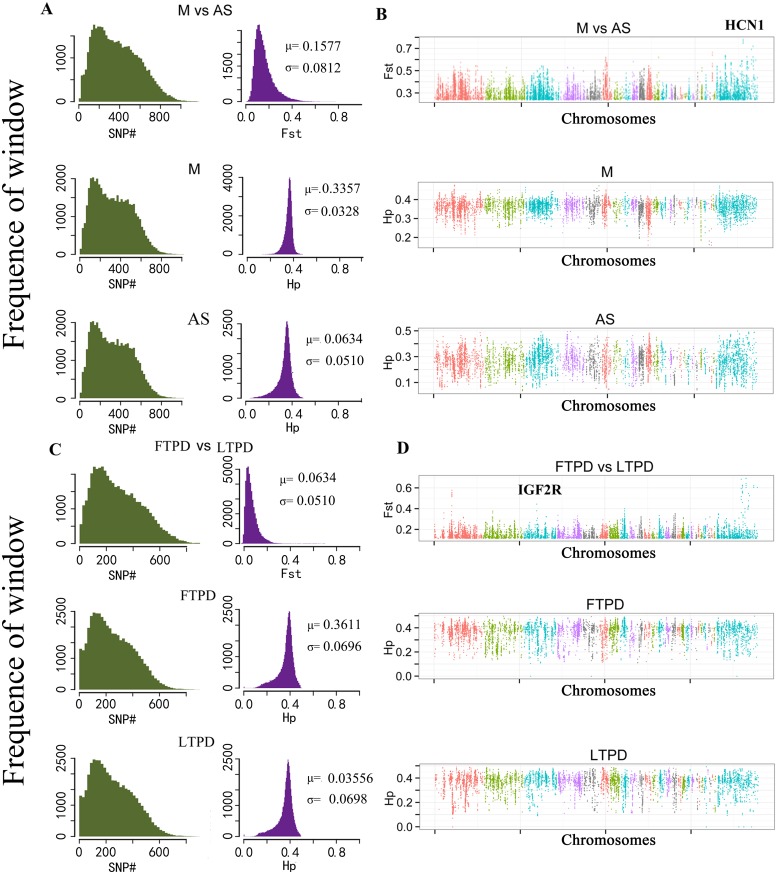
Genomic regions associated with artificial selection. (**A) and (C)**, Distribution of window number, fixation index (Fst), and heterozygosity (Hp) for all 40-Kb windows. Bins of Fst and Hp are presented along the x axes. μ, mean; σ, standard deviation; M, mallard population; FTPD, fat-type Pekin duck population; LTPD, lean-type Pekin duck population; CMD, China Micro-duck population; AS, combined populations of FTPD, LTPD and CMD. (**B) and (D)**, The positive end of the Fst distribution and the negative end of the Hp distribution plotted along duck pseudo-chromosomes 1–15, 16–28 and pseudo-chromosome UN (pseudo-chromosomes are separated by colors). A window with its Fst value falling into the top 200 highest Fst values and at least one of the two Hp values in the compared groups (M-AS or FTPD-LTPD) falling into the smallest 400 Hp values is considered as a selected window.

Selective sweeps occur when beneficial genetic variants increase in frequency due to positive selection [11]. In addition, positive selection always leads to reduced heterozygosity of the selected population and increased differentiation between populations around the selected site [10]. Therefore, genomic regions under selection can be identified based on reduced heterozygosity or increased differentiation in the selected populations. In this study, regions under selection between the LTPD and FTPD populations, as well as the M and AS populations were investigated by identifying regions with an increased fixation index (Fst) and reduced pooled heterozygosity (Hp) [25] using a 40Kb-sliding window (step = 20Kb). The distribution of observed Fst and Hp are shown in Fig 2A–2D for both comparisons. In total, 133 windows (76 continuous regions) overlapping 78 genes were found to be under selection between the M and AS populations (S7 Table), while 134 windows (76 continuous domains) overlapping 99 genes were found to be under selection between the FTPD and LTPD populations (S8 Table).

**Fig 3:**
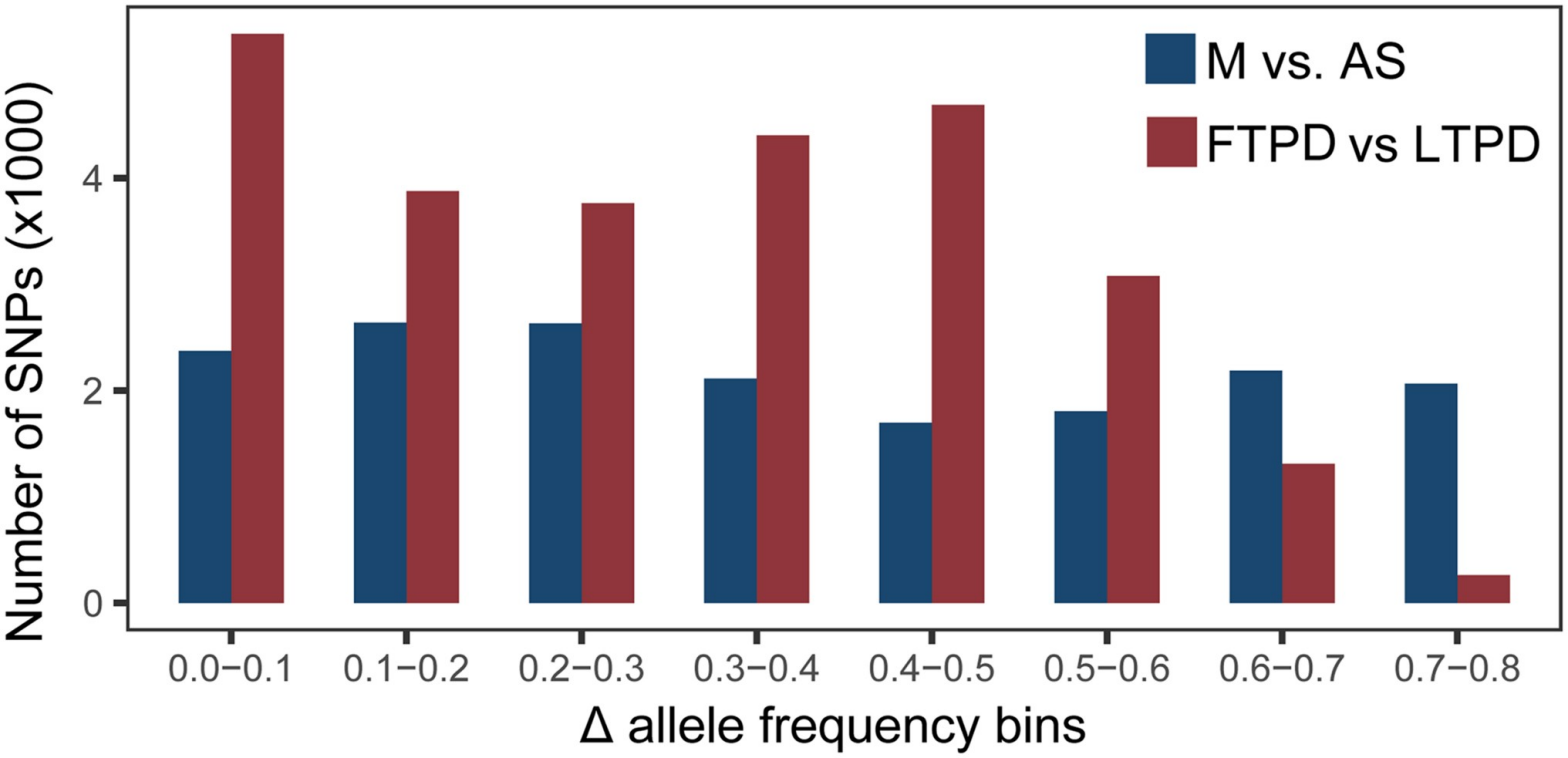
Absolute allele frequencies (ΔAF) for SNPs under selection between M and AS and between the FTPD and LTPD populations. Bins of ΔAF are presented along the x axis. The number of SNPs present in each bin is presented along the y axis.

Given the comprehensive sampling in our study and the correlation in allele frequencies amongst the populations studied, highly differentiated SNPs are likely to have either been directly targeted by selection or occurred in the vicinity of loci under selection. Therefore, we calculated the absolute allele frequency (ΔAF) for each SNP located in regions under selection and sorted them into 10% bins (i.e. ΔAF = 0.00 to 0.10, 0.10 to 0.20, etc.) (Fig 3, S1 and S2 Data). Of the 19,100 SNPs located in regions under selection between the AS and M populations, 9,761 displayed low ΔAF (≤ 0.40) and 9,339 displayed high ΔAF (≥ 0.41). Notably, ~1% (1,580) of identified SNPs were fixed or nearly fixed in one group (ΔAF ≥ 0.81). A much higher number of SNPs (26,784) were located in regions under selection in FTPD and LTPD comparison compared to that in M and AS comparison, however, only 35 of them were fixed or nearly fixed in one group (ΔAF ≥ 0.81). These results suggest that although the artificial selection imposed on the FTPD and LTPD populations resulted in a high number of selected SNPs, the majority of detected SNPs were not fixed over the relatively short time span (~ 40 years).

### Genes detection located in artificial selection

To detect genes targeted by artificial selection in ducks, we identified genes under selection between the M and AS populations and the LTPD and FTPD populations. In total, 78 genes were located in regions under selection between the M and AS populations (S7 Table), many of which are involved in morphology and physiology. Two of the selected genes (MITF and LYST) are known to be crucial for feather coloring [26–29]. Selected genes involved in biosynthetic processes include PTGS2, a key enzyme in prostaglandin biosynthesis that inhibits female reproductive processes when disrupted in mice [30], IGF1R, which results in growth retardation when mutated in humans [31, 32], and MEF2A, which induces myogenic development and is involved in skeletal muscle regeneration [33, 34]. GO analysis was carried out to identify the functional role of genes under selection. GO terms enriched for genes under selection between the M and AS populations were mainly involved in pigmentation (4 of the top 10 terms, P-value = 4.2×10^−3^ to 5.6 ×10^−3^) and biosynthetic processes (4 of the top 10 terms, P-value = 2×10^−4^ to 9×10^−4^) (Table 3 and S9 Table), indicating that feather color and substance synthesis have been strongly selected during the artificial selection process.

**Table 3:** TOP 10 GO terms enriched for selected genes.

GO Term	P-value	Adjusted p-values	Gene counts
Enriched for genes under selection between the M and AS populations			
Melanocyte differentiation	0.0002	0.003	3
Endosome transport via multivesicular body sorting pathway	0.0002	0.003	2
Pigmentation	0.0004	0.004	4
Pigment cell differentiation	0.0005	0.004	3
Developmental pigmentation	0.0009	0.0058	3
Nucleus organization	0.0041	0.0066	3
Cellular biosynthetic process	0.0042	0.0066	26
Fatty acid derivative biosynthetic process	0.0049	0.0066	2
Icosanoid biosynthetic process	0.0049	0.0066	2
Organic substance biosynthetic process	0.0056	0.0066	26
Enriched for genes under selection between the FTPD and LTPD populations			
Detection of stimulus involved in sensory perception	0.0013	0.0086	4
Regulation of fat cell differentiation	0.0015	0.0086	4
Detection of chemical stimulus involved in sensory perception of smell	0.0017	0.0086	4
Positive regulation of fat cell differentiation	0.002	0.0086	3
Fat cell differentiation	0.0021	0.0086	5
Detection of chemical stimulus involved in sensory perception	0.0025	0.0086	4
Positive regulation of myoblast differentiation	0.0026	0.0086	3
Positive regulation of NIK/NF-kappab signaling	0.0031	0.0086	2
Sensory perception of smell	0.0032	0.0086	4
Response to muscle stretch	0.0037	0.0086	2

Note: Enriched terms are color-coded to reflect relatedness in ontology or functional proximity. Blue, pigmentation; yellow, biosynthetic processes; green, sensory perception; grey, fat deposition and muscle development. For each term, gene counts indicate number of genes overlapping with selected regions.

LTPD and FTPD have been produced through artificial selection of ducks with higher breast muscle percentage or higher carcass fatness percentage since the 1990s, respectively [20]. In this study, 99 genes were found to be under selection between the FTPD and LTPD populations (S8 Table), 16 of which are involved in skeletal muscle development and fat deposition (S8 Table). GO analysis of the genes under selection between the FTPD and LTPD populations confirmed enrichment of genes involved in skeletal muscle development and fat deposition. Six out of top 10 terms were involved in fat deposition and muscle development (P-value = 1.3×10^−3^ to 3.7 ×10^−3^). The remained four terms were related with sensory perception (P-value = 1.5×10^−3^ to 3.2 ×10^−3^) (Table 3 and S10 Table), suggesting differences sense of smell between the FTPD and LTPD populations.

### *HCN1* is associated with reduced heart rates in AS populations

**Fig 4:**
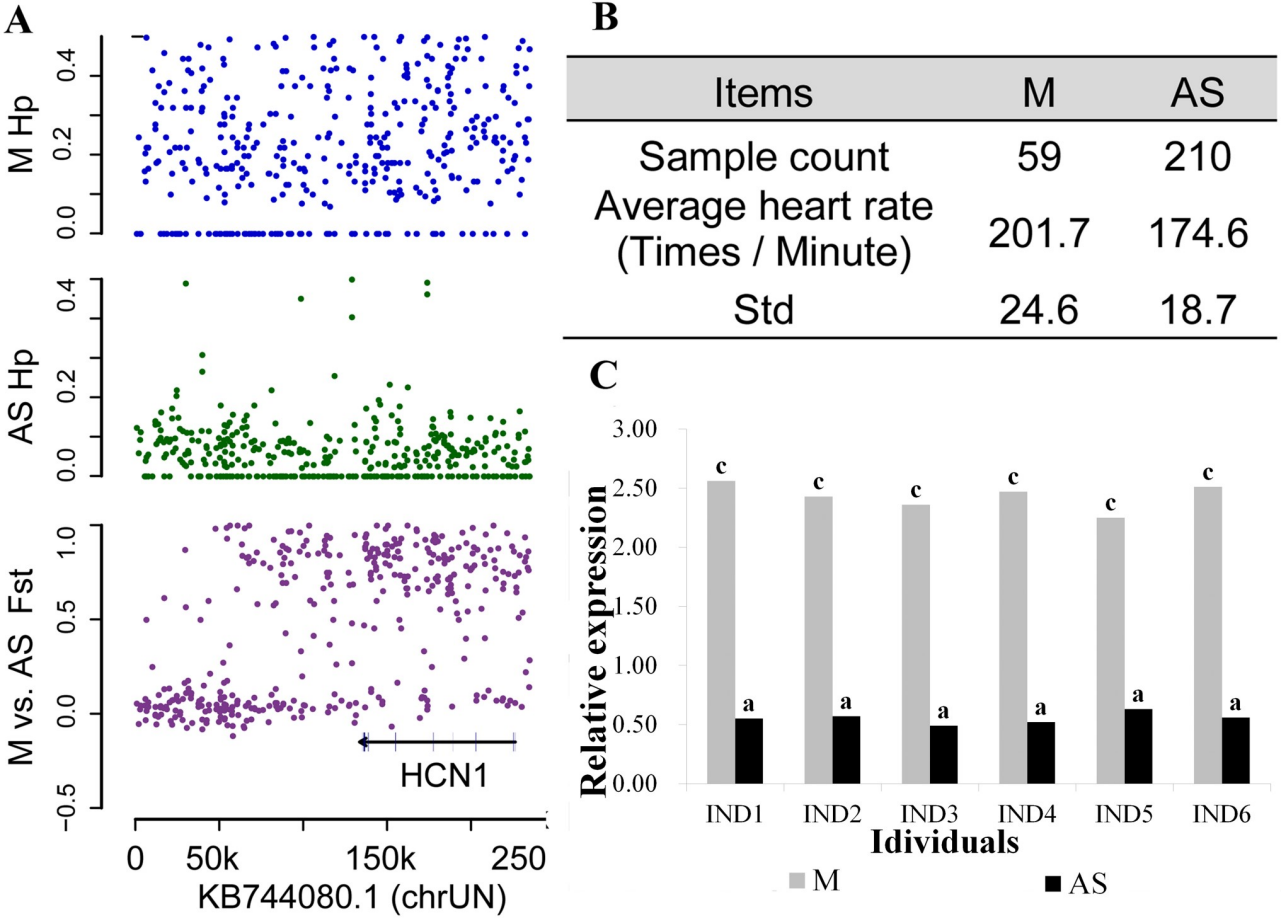
Artificial selection is responsible for reduced heart rates in AS populations. (**A)**, Fixation index (Fst) and heterozygosity (Hp) for single SNPs in scaffold KB744080.1. *HCN1* was identified as the gene under the strongest selective pressure between the M and AS populations and is located on scaffold KB744080.1 (see Fig 2B and S2 Table). (**B)**, Average heart rates in M and AS populations; Std, estimated standard deviation. **(C)**, *HCN1* mRNA expression levels in the heart tissue of M and AS ducks; **a, c**, extremely significant difference.

It had been reported that HCN1 is expressed in the sinoatrial node [35–37] and contributes to stable heart rates in mice [38]. In this study, HCN1 was identified as the gene under the strongest selective pressure (Fst = 0.78) between the M and AS populations with a lower Hp value (0.14) in the AS population (Figs 2A, 2B, and 4A and S11 Table). This result suggests significant differences in heart rates may exist between M and AS ducks. Therefore, we compared the heart rates of M (n = 59) and AS (n = 210) ducks, identifying a significantly higher average heart rate in M (201.70 beats/minute) compared to AS (174.60 beats/minute; p-value = 0.00247) (Fig 4B). Although no nonsynonymous mutations were identified in the HCN1 gene, a two base (GC) deletion at base 1,357 of HCN1 resulted in expression of a splice variant in the AS population (S12 Table). In addition, M ducks displayed higher HCN1 mRNA expression level compared to AS ducks (n = 6/group) (Fig 4C). These results indicate that artificial selection resulted in reduced *HCN1* expression is likely responsible for the reduced heart rates observed in AS compared to M ducks.

### *IGF2R* is associated with increased lean meat ratios in LTPD populations

As a result of artificial selection, the percentage and thickness of breast muscle has significantly increased, while the skin fat percentage has significantly decreased in LTPD compared to FTPD (Table 4, S1 Table and S2 Fig). These differences indicate that selective pressure exerted on LTPD and FTPD populations during artificial selection has had significant effects on skeletal muscle development and fat deposition.

**Table 4:** The comparison of slaughter performances between LTPD and FTPD.

Tissues	Male		Female	
	FTPD	LTPD	FTPD	LTPD
Body weight/g	2764^a^±124.3	3360^c^±143	2679^a^±69	3313^c^±226
Breast muscle weight/g	177^a^±26.2	394^c^±44	186^a^±37	438^c^±51
Leg muscle weight/g	116^a^±13.6	294^c^±48	196^a^±11	300^c^±20
Skin fat weight	524^a^±51.6	535^a^±51	551^a^±39	524^a^±99
Breast muscle rate/%	8.83^a^±1.0	15.4^c^±1.3	9.53^a^±1.7	17.3^c^±1.4
Leg muscle rate/%	11.6^a^±1.1	11.5^a^±1.6	11.42^a^±1.0	11.8^a^±1.3
Skin fat rate/%	19.0^a^±2.2	20.1^a^±1.7	28.26^b^±1.7	20.7^a^±3.2

Note: interphase lowercase letter superscripts for the same trait within male or female mean extremely significant difference; adjacent lowercase letter superscripts for the same trait within male or female mean significant difference; same lowercase letter superscripts for the same trait within male or female mean no significant difference

**Fig 5:**
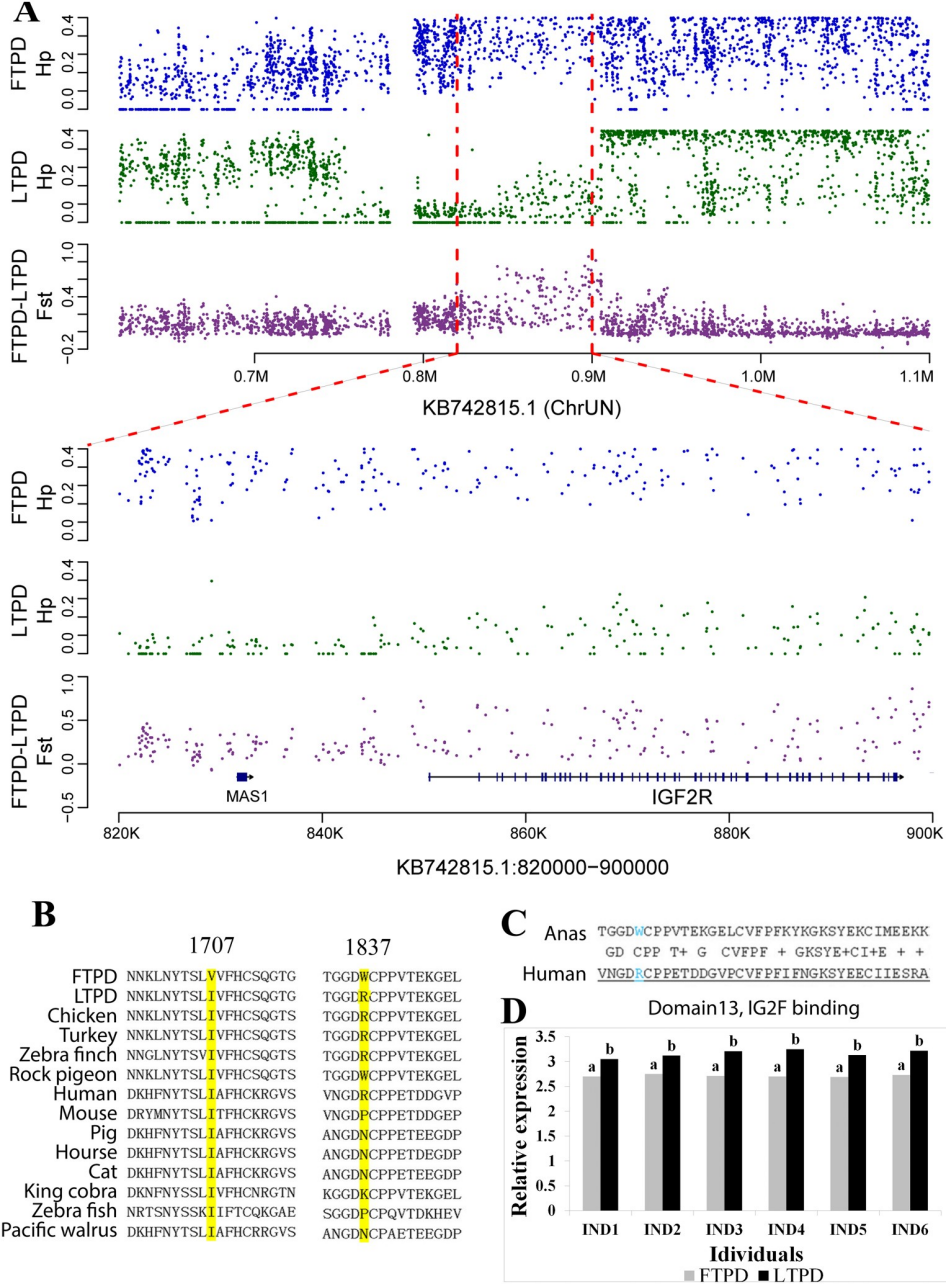
*IGF2R* is responsible for the increased lean meat percentage observed in the LTPD population. (**A)**, Fixation index (Fst) and heterozygosity (Hp) for single SNPs in scaffold KB742815.1. *IGF2R* was identified as the gene under selection between the LTPD and FTPD populations and is located on scaffold KB742815.1. (**B)**, Multiple sequence alignment of the IGF2R protein across 14 species at the 1,707 and 1837 amino acid positions. (**C)**, Comparison of the 13th extracellular repeat region of the IGF2R protein between humans and ducks. **(D)**, *IGF2R* mRNA expression levels in FTPD and LTPD skeletal muscle.

*IGF2* plays a crucial role in muscle mass development in pigs [39] and in mice [40]. In addition, previous studies have indicated *IGF2R*, a negative regulator of *IGF2* [41], plays a role in determining lean meat ratios [42]. Therefore, we investigated relationship between *IGF2R* and differential lean meat ratios and fat percentages observed between LTPD and FTPD populations. In this study, *IGF2R* was found to be under selection between the LTPD and FTPD populations (Fst = 0.39, HP(FTPD) = 0.45, Hp(LTPD) = 0.21) (Figs 2C, 2D, and 5A and S13 Table). Four SNPs resulting in three amino acid substitutions were identified within the *IGF2R* gene (S14 Table). The first nonsynonymous mutation was a 241A>G substitution resulting in an Ile81Val substitution in the duck IGF2R protein. Because this Ile81Val substitution is not located in a conserved or functional region, we did not investigate this site further. The second nonsynonymous mutation identified was a 5119A>G mutation leading to a Val1707Ile substitution in the IGF2R protein. A Val1707Ile substation located in the highly conserved CIMR region of IGF2R was also identified based on comparison of the duck IGF2R protein sequence with the NCBI conserved domain database (http://www.ncbi.nlm.nih.gov/Structure/cdd/wrpsb.cgi) (S3 Fig) [43]. This Val1707Ile amino acid substitution was observed in the LTPD population and is highly conserved across 14 species (Fig 5C). Due to codon degeneracy, the third (5509T>C) nonsynonymous mutation combined with the fourth (5511G>A) nonsynonymous mutation resulted in a Trp1837Arg substitution in the LTPD population. Multi-species protein sequence alignments revealed the Arg substitution observed in LTPD is also highly conserved in the majority of bird species profiled to date (Fig 5B). Studies in humans have demonstrated that IGF2R is a transmembrane receptor molecule with a large extracellular domain comprised of 15 repeat regions and a small intracellular region. The 13th extracellular repeat region is responsible for regulating the binding affinity of IGF2R to IGF2 (Fig 5C) [44, 45]. Based on comparison of the duck and human IGF2R protein sequences, the Trp1837Arg substitution was found to be located in the 13^th^ extracellular repeat region of the duck IGF2R protein (Fig 5C). Thus, this amino acid substitution is likely responsible for functional differences leading to increased lean meat ratios in LTPD compared to FTPD populations.

In order to confirm the presence of two of the nonsynonymous mutations (5119A>G, and 5509T>C) in the *IGF2R* gene, which are likely responsible for the differential phenotypes observed between LTPD and FTPD populations, we cloned and sequenced the relevant regions of the *IGF2R* gene (S4–S7 Figs). In addition, we also compared the allele frequencies at the two SNP sites. At the 5119A>G mutation site, the G allele frequency was higher in the LTPD than in FTPD population (0.9130 and 0.2955, respectively) (S15 Table and S4 Fig), indicating near fixation of the G allele in the LTPD population. For the 5509T>C mutation, the T allele frequency was higher in the LTPD than in FTPD population (0.8913 and 0.2727, respectively) (S16 Table and S5 Fig).

To decipher whether the candidate mutations affect gene expression, we examined *IGF2R* mRNA expression level in the breast muscle of FTPD and LTPD populations. The result showed IGF2R mRNA level was significantly higher in breast muscle of FTPD compared to LTPD populations (Fig 5D). These results indicate that artificial selection has resulted in selection for *IGF2R* mutations and differential IGF2R protein levels in breast muscle between LTPD and FTPD populations. Previous studies have identified mutations in crucial genes leading to phenotypic differences between animal populations, such as the *MGAM* gene in dogs, which catalyzes the hydrolysis of maltose to glucose, and the *MITF* and *KIT* genes in dogs and pigs, which affect coat color [10, 46]. Therefore, the mutations observed between populations in this study indicate *IGF2R* is a candidate gene for regulation of skeletal muscle mass in ducks.

Whole genome sequencing using pooled or individual samples provides an extremely powerful approach for detecting genetic differences associated with phenotypic traits in animals [47]. With this approach, diverse genetic signatures of domestication and evolution have been elucidated in a number of animal species [8–13]. In ducks, muscle growth and lipid deposition are two important features representing the main breeding objectives. Previously, 10 and 11 candidate genes related to duck muscle growth and lipid deposition have been reported [48]. However, the selected genes identified in this study do not overlapped with these previously reported candidate genes. As the previous study was performed using different duck breeds (a native Pekin duck with higher fat content and a Pekin duck bred crossbred with a native British duck breed with higher lean meat percentage and intramuscular fat content) [48,49], the differences observed between these studies suggest differential adaption mechanisms underlie the same phenotypic changes observed across distinct duck populations.

In conclusion, the results of this study broaden our knowledge of the effects of artificial selection on the duck genome, shedding light on the allelic variation underlying relevant production traits across industrially relevant duck populations. These results will enable the future improvement of duck breeding schemes and support further investigation of the mechanisms underlying artificial selection in ducks.

## Materials and methods

### Anchoring duck scaffolds to pseudo-chromosomes

We utilized the chicken (*Gallus gallus*, Ensembl version: 4.76) and turkey (*Meleagris gallopavo*, Ensembl version: UMD2.76) chromosomal assemblies to anchor the duck scaffolds to pseudo-chromosomal sequences. We first performed whole genome alignment of duck scaffolds longer than 2 kb with harboring at least two protein-coding genes against chicken chromosome sequences using the promer program in the MUMmer 3.0 package [50]. We then filtered the alignments using delta-filter, with the–g parameter enabled to filter for 1-to-1 global alignments without rearrangements. Alignments with an identity < 30 and alignment uniqueness (percent of the alignment matching to unique reference and query sequences) < 50 were discarded. The resulting alignments indicating ambiguous order and orientation of duck scaffold sequences were visualized as dot plots for manually checking. The same methods were used to align scaffolds to the turkey chromosomal assembly. The anchored pseudo-chromosomes were finalized by discarding scaffolds with inconsistent ordering or orientation when aligned to the chicken and turkey chromosomes. In total, 1,141 scaffolds were anchored onto 28 pseudo-chromosomes.

### Sample preparation

All protocols of birds handling and sampling were approved by the Animal Care and Use Committee of Chinese Academy of Agricultural Sciences (CAAS), and all efforts were made to minimize the suffering of animals according to recommendations proposed by the European Commission (1997). The study was carried out in accordance with the approved protocol. All methods were conducted in accordance with relevant guidelines. Birds were slaughtered using the electric shock method followed by jugular vein bloodletting method within 30 seconds to ameliorate their suffering. LTPD, FTPD, CMD, and M duck populations were used to collect venous blood for genomic DNA extraction in this study. LTPD, FTPD, and CMD populations developed by the Institute of Animal Science, Chinese Academy of Agricultural Sciences were sampled. Mallard (M), a widely accepted ancestor of domesticated ducks [18], raised by the Ji’ao Austrian Agricultural Science and Technology Co., Ltd in Fenghua City, Zhejiang Province, was also sampled. For each population, we randomly selected 30 healthy adult birds and collected blood from the wing vein to extract DNA as previously described [51]. DNA from each individual was mixed in equimolar ratios to generate a DNA pool for sequencing. Additional DNA from each individual was used to clone genes crucial for relevant production traits.

### Library construction and sequencing

Paired-end sequencing libraries were generated for each pool using standard Illumina sequencing protocols. The constructed libraries were sequenced as 100 bp paired-end reads on an Illumina HiSeq 2000, resulting in ~ 40x coverage per pool (S3 Table).

### Read mapping

We used the Burrows-Wheeler Alignment tool (BWA) [52] with default settings to map the raw reads to the duck genome assembly (ensemble version: BGI_duck_1.076), resulting in an alignment rate of nearly 90% per pool (S4 Table). We sorted the alignments and removed PCR duplicates using the picard-tools MarkDuplicates.jar package (http://picard.sourceforge.net/). To improve the alignment accuracy, we performed ‘multiple sequence realigning’ around putative INDEL regions using the RealignerTargetCreator/IndelRealigne programs in the Genome Analysis Toolkit (GATK, http://www.broadinstitute.org/gatk/). The remained alignments were used for downstream sequence variation analysis.

### SNP and INDEL detection and annotation

We used a combination of GATK [53] and freebayes [54] to detect SNPs and INDELs. First, GATK and freebayes were run using default settings independently to produce a pair of raw calling sets. Putative variants from freebayes with a QUAL score < 30 were discarded. We then applied stringent parameters to filter and integrate the two sets of results. Due to the distinct quality properties of SNPs and INDELs, we applied different filtering criteria for each. For SNPs, SelectVariants in GAKT was used to identify SNPs consistently called between GATK and freebayes. Consistently called SNPs were subjected to a hard filter step using the parameters recommended by the GATK mentor: QD < 2.0 || FS > 60.0 || MQ < 40.0 || MappingQualityRankSum < -12.5 || ReadPosRankSum < -8.0.3. Variants with ultra-high (> 500) or ultra-low (< 10) coverage were discarded. Finally, bi-allelic variants with allele frequency greater than 0.05 were included in the final set of variants. For INDELs, SelectVariants in GAKT was used to identify INDELs consistently called between GATK and freebayes. Consistently called INDELs were subjected to a hard filter step using the parameters recommended by the GATK mentor: QD < 2.0 || FS > 200.0 || ReadPosRankSum < -20.0.3. Variants with ultra-high (> 500) or ultra-low (< 10) coverage were further discarded. Finally, bi-allelic variants shorter than 5 bp and an allele frequency greater than 0.05 were included in the final set of variants.

To explore the location and effect of sequence variants on gene transcription and functionality, the identified SNPs and INDELs were annotated against the reference genome annotation using snpEff [55].

### Analysis of selective sweeps, genes under selection, and variants harbored by selected genes

We used allele counts at SNP positions of sliding windows (bin size, 40 Kb; step size, 20 Kb) to identify signatures of selection. To reduce the number of false positives, windows contained > 10 SNPs were used in downstream analysis. For each comparison between two pools of sequencing data, we calculated Fst using popoolation2 [56] and Hp according to [9]. A window was defined as a potentially selected window used in downstream analysis if it simultaneously met the following two criteria: (i) Fst value of the window should fall into the top 200 highest Fst values among all the windows across the duck genome, and (ii) Hp value of the window should fall into the smallest 400 Hp values in at least one duck population in the two compared groups (M-AS or FTPD-LTPD). Genes overlapping with those windows were defined as genes under selection. We then calculated FST and a pooled heterozygosity score, Hp, at individual variant sites. In order to identify variants associated with selective sweeps, we manually analyzed the SNPs and INDELs located in coding regions of genes under selection. We focused on variants: 1) in genes under selection; 2) resulting in nonsynonymous amino acid changes or nonsense mutations; and 3) were evolutionary conserved. The conversed positions of each analyzed protein sequence were retrieved from multiple protein sequence alignments calculated by MAFFT [57]. We also analyzed all the aforementioned variations using PROVEAN (http://provean.jcvi.org/index.php), which predicts the impact of mutations on the biological function of proteins based on conservation analysis of homologs automatically searched for in the NCBI Nr database.

### GO analysis of selected genes

We used the duck Gene Ontology (GO) annotation information from the Ensembl Genome Browser to perform enrichment analysis of genes under selection. For each query, we tested over represented GO terms against a background (the whole genome set of protein-coding genes in the annotation) using the GOstats Bioconductor R package (https://www.r-project.org/) [58].

### The alignment of multiple protein sequences of IGF2R proteins

To investigate the conservation of amino acid substitutions occurring at amino acid positions 1,707 and 1,837 of the duck IGF2R protein, we obtained the duck IGF2R protein sequences of FTPD and LTPD. We then downloaded the IGF2R protein sequences deriving from 12 additional species. The online multiple protein sequence alignment tool, COBALT (www.ncbi.nlm.nih.gov/tools/cobalt/) [59], was used to identify conserved amino acids at positions 1,707 and 1,837 of the duck IGF2R protein.

### Validation of *IGF2R* SNPs

PCR amplification and DNA sequencing approaches were used to validate the two SNPs harbored by the duck *IGF2R* gene (5119A>G, and 5509T>C). The two primer pairs used are listed in S17 Table. The total reaction volume for each PCR reaction was 20 μL, containing 10 μL of 2×Premix Taq PCR Solution, 0.7 μL of each primer (S17 Table), 1 μL of normalized template cDNA, and 7.6 μL ultrapure water. PCRs were performed as follows: 1 cycle of denaturation at 94°C for 5 min, 36 cycles of denaturation at 94°C for 30 s, annealing at 50°C for 40 s, extension at 72°C for 2 min, and a final extension at 72°C for 10 min. The PCR products were separated using 1.2% agarose gel electrophoresis, and the target fragments were retrieved and purified by the EZgene Gel/PCR Extraction Kit (Biomiga, Shanghai, China) for DNA sequencing.

### Detection of *IGF2R* and *HCN1* mRNA expression

qRT-PCR was used to detect *IGF2R* and *HCN1* mRNA expression levels and the corresponding primers are presented in S18 Table. qRT-PCR was performed using the SYBR PrimeScript RT-PCR Kit (TaKaRa, Dalian, China) with SYBR Green dye as described previously [60]. Briefly, qRT-PCR reactions were carried out with an iCycler IQ5 Multicolor Real-Time PCR Detection System (Bio-Rad, USA). The qRT-PCR reaction volume was 25 μL, contained 1 μL of cDNA template, 12.5 μL of SYBR Premix ExTaq, 9.5 μL of sterile water, and 1 μL of each gene-specific primer. Thermal cycling parameters were 1 cycle at 95°C for 2 min, 40 cycles of 95°C for 15 s, and 60°C for 34 s. Dissociation curve analysis was done after each real-time reaction to ensure that there was only one product. The qRT-PCR analysis of each sample was done in triplicate.

## Supporting information

S1 Fig Phylogenetic tree of the four duck populations. (TIF)

S2 Fig Breast muscle thickness comparison between FTPD and LTPD. **a**, The breast muscle thickness of LTPD. **b**, The breast muscle thickness of FTPD.
(TIF)

S3 Fig The 1,707th amino acid of duck IGF2R located in the CIMR conservation region. (TIF)

S4 Fig The confirmation of nucleotide 5119A>G in IGF2R gene. All of “A” at this site were marked in blue.
(TIF)

S5 Fig The confirmation of nucleotide 5509T>C in IGF2R gene. All of “C” at this site were marked in blue.
(TIF)

S6 Fig The confirmation of Val1707Ile substitution. “V” and “I” at this site were marked in blue.
(TIF)

S7 Fig The confirmation of Trp1837Arg substitution. “W” and “R” at this site were marked in blue.
(TIF)

S1 Table Comparison of four duck populations studied in this paper (Values are means ± s.d). (DOCX)

S2 Table The results of anchoring duck scaffolds to psuo-chromosomes. (DOCX)

S3 Table Pool information for resequencing data from four duck populations. (DOCX)

S4 Table Basic sequenced data statistics for the four duck population. (DOCX)

S5 Table Identified SNPs for each duck population. (DOCX)

S6 Table Identified Indels for each duck population. (DOCX)

S7 Table The genes under selection between artificial selection populations and their ancestor. (DOCX)

S8 Table The genes under selection between LTPD and FTPD. (DOCX)

S9 Table The functional enrichment analysis for genes under selection between M and AS. (DOCX)

S10 Table The functional enrichment analysis for genes under selection between FTPD and LTPD. (DOCX)

S11 Table The top 20 selected genes in the M-AS comparison. (DOCX)

S12 Table The annotation of Indels identified in the M-AS comparison. (DOCX)

S13 Table The top 20 selected genes in the FTPD-LTPD comparison. (DOCX)

S14 Table The annotation of SNPs by *IGF2R* harbored missense variants. (DOCX)

S15 Table The allele frequencies of G and A at 5119A>G mutation site in the LTPD and FTPD populations. (DOCX)

S16 Table The allele frequencies of T and C at 5509T>C mutation site in the LTPD and FTPD populations. (DOCX)

S17 Table Primers used for validation the SNPs in the duck IGF2R gene. (DOCX)

S18 Table Primers used for analyzing mRNA expression levels of *IGF2R* and *HCN1*. (DOCX)

S1 Data The absolute allele frequency (ΔAF) between M and AS. (XLSX)

S2 Data The absolute allele frequency (ΔAF) between FTPD and LTPD. (XLSX)

## References

[1] Fisher RA. The Genetic Theory of Natural Selection. Oxford University of Press; 1930.

[2] Schiavo G, Galimberti G, Calo DG, Samore AB, Bertolini F, Russo V, et al Twenty years of artificial directional selection have shaped the genome of the Italian Large White pig breed. Anim Genet. 2016;47(2):181–91. 10.1111/age.12392 . 26644200

[3] Wang YM, Xu HB, Wang MS, Otecko NO, Ye LQ, Wu DD, et al Annotating long intergenic non-coding RNAs under artificial selection during chicken domestication. BMC Evol Biol. 2017;17(1):192 10.1186/s12862-017-1036-6 . 28810830 PMC5558714

[4] Kim ES, Cole JB, Huson H, Wiggans GR, Van Tassell CP, Crooker BA, et al Effect of artificial selection on runs of homozygosity in u.s. Holstein cattle. PLoS One. 2013;8(11):e80813 10.1371/journal.pone.0080813 . 24348915 PMC3858116

[5] Loehr J, Carey J, Hoefs M, Suhonen J, Ylonen H. Horn growth rate and longevity: implications for natural and artificial selection in thinhorn sheep (Ovis dalli). J Evol Biol. 2007;20(2):818–28. 10.1111/j.1420-9101.2006.01272.x . 17305848

[6] Atanur SS, Diaz AG, Maratou K, Sarkis A, Rotival M, Game L, et al Genome sequencing reveals loci under artificial selection that underlie disease phenotypes in the laboratory rat. Cell. 2013;154(3):691–703. 10.1016/j.cell.2013.06.040 . 23890820 PMC3732391

[7] Murray G, Soares A, Novak BJ, Schaefer NK, Cahill JA, Baker AJ, et al Natural selection shaped the rise and fall of passenger pigeon genomic diversity. Science, 2017; 358(6365): 951–954. 10.1126/science.aao0960 29146814

[8] Groenen MA, Archibald AL, Uenishi H, Tuggle CK, Takeuchi Y, Rothschild MF, et al Analyses of pig genomes provide insight into porcine demography and evolution. Nature. 2012;491(7424):393–8. 10.1038/nature11622 . 23151582 PMC3566564

[9] Rubin CJ, Zody MC, Eriksson J, Meadows JR, Sherwood E, Webster MT, et al Whole-genome resequencing reveals loci under selection during chicken domestication. Nature. 2010;464(7288):587–91. 10.1038/nature08832 . 20220755

[10] Axelsson E, Ratnakumar A, Arendt ML, Maqbool K, Webster MT, Perloski M, et al The genomic signature of dog domestication reveals adaptation to a starch-rich diet. Nature. 2013;495(7441):360–4. 10.1038/nature11837 . 23354050

[11] Carneiro M, Rubin CJ, Di Palma F, Albert FW, Alfoldi J, Barrio AM, et al Rabbit genome analysis reveals a polygenic basis for phenotypic change during domestication. Science. 2014;345(6200):1074–9. 10.1126/science.1253714 . 25170157 PMC5421586

[12] Liu S, Lorenzen ED, Fumagalli M, Li B, Harris K, Xiong Z, et al Population genomics reveal recent speciation and rapid evolutionary adaptation in polar bears. Cell. 2014;157(4):785–94. 10.1016/j.cell.2014.03.054 . 24813606 PMC4089990

[13] Burga A, Wang W, Ben-David E, Wolf PC, Ramey AM, Verdugo C, et al A genetic signature of the evolution of loss of flight in the Galapagos cormorant. Science. 2017;356(6341). 10.1126/science.aal3345 . 28572335 PMC5567675

[14] Hackett SJ, Kimball RT, Reddy S, Bowie RC, Braun EL, Braun MJ, et al A phylogenomic study of birds reveals their evolutionary history. Science. 2008;320(5884):1763–8. 10.1126/science.1157704 . 18583609

[15] Duggan BM, Hocking PM, Schwarz T, Clements DN. Differences in hindlimb morphology of ducks and chickens: effects of domestication and selection. Genet Sel Evol. 2015;47:88 10.1186/s12711-015-0166-9 . 26576729 PMC4647608

[16] Senapati MR, Behera PC, Maity A, Mandal AK. Comparative histomorphological study on the thymus with reference to its immunological importance in quail, chicken and duck. Explor Anim Med Res. 2015;5(1):5.

[17] Radzimirska M. Morphology, topography and morphometrical analysis of the mandibular ganglion in domestic duck and domestic Turkey2010. 405–9 p.

[18] Li HF, Zhu WQ, Song WT, Shu JT, Han W, Chen KW. Origin and genetic diversity of Chinese domestic ducks. Mol Phylogenet Evol. 2010;57(2):634–40. 10.1016/j.ympev.2010.07.011 . 20674751

[19] Huang Y, Li Y, Burt DW, Chen H, Zhang Y, Qian W, et al The duck genome and transcriptome provide insight into an avian influenza virus reservoir species. Nat Genet. 2013;45(7):776–83. 10.1038/ng.2657 . 23749191 PMC4003391

[20] Xu T, Gu L, Schachtschneider KM, Liu X, Huang W, Xie M, et al Identification of differentially expressed genes in breast muscle and skin fat of postnatal Pekin duck. PLoS One. 2014;9(9):e107574 10.1371/journal.pone.0107574 . 25264787 PMC4180276

[21] Zhang G, Li C, Li Q, Li B, Larkin DM, Lee C, et al Comparative genomics reveals insights into avian genome evolution and adaptation. Science. 2014;346(6215):1311–20. 10.1126/science.1251385 . 25504712 PMC4390078

[22] Wojcik E, Smalec E. Description of the mallard duck (Anas platyrhynchos) karyotype. Folia Biol (Krakow). 2007;55(3–4):115–20. . 18274254 10.3409/173491607781492588

[23] Rubin CJ, Megens HJ, Martinez Barrio A, Maqbool K, Sayyab S, Schwochow D, et al Strong signatures of selection in the domestic pig genome. Proc Natl Acad Sci U S A. 2012;109(48):19529–36. 10.1073/pnas.1217149109 . 23151514 PMC3511700

[24] Lee TH, Guo H, Wang X, Kim C, Paterson AH. SNPhylo: a pipeline to construct a phylogenetic tree from huge SNP data. BMC Genomics. 2014; 15(1):162 10.1186/1471-2164-15-162 24571581 PMC3945939

[25] Konkel MK, Walker JA, Hotard AB, Ranck MC, Fontenot CC, Storer J, et al Sequence Analysis and Characterization of Active Human Alu Subfamilies Based on the 1000 Genomes Pilot Project. Genome Biol Evol. 2015;7(9):2608–22. 10.1093/gbe/evv167 . 26319576 PMC4607524

[26] Goding CR. Mitf from neural crest to melanoma: signal transduction and transcription in the melanocyte lineage. Genes Dev. 2000;14(14):1712–28. . 10898786

[27] Tachibana M. MITF: a stream flowing for pigment cells. Pigment Cell Res. 2000;13(4):230–40. . 10952390 10.1034/j.1600-0749.2000.130404.x

[28] Gutierrez-Gil B, Wiener P, Williams JL. Genetic effects on coat colour in cattle: dilution of eumelanin and phaeomelanin pigments in an F2-Backcross Charolais x Holstein population. BMC Genet. 2007;8:56 10.1186/1471-2156-8-56 . 17705851 PMC1994163

[29] Runkel F, Bussow H, Seburn KL, Cox GA, Ward DM, Kaplan J, et al Grey, a novel mutation in the murine Lyst gene, causes the beige phenotype by skipping of exon 25. Mamm Genome. 2006;17(3):203–10. 10.1007/s00335-005-0015-1 16518687

[30] Lim H, Paria BC, Das SK, Dinchuk JE, Langenbach R, Trzaskos JM, et al Multiple female reproductive failures in cyclooxygenase 2-deficient mice. Cell. 1997;91(2):197–208. . 9346237 10.1016/s0092-8674(00)80402-x

[31] Abuzzahab MJ, Schneider A, Goddard A, Grigorescu F, Lautier C, Keller E, et al IGF-I receptor mutations resulting in intrauterine and postnatal growth retardation. N Engl J Med. 2003;349(23):2211–22. 10.1056/NEJMoa010107 . 14657428

[32] Lei M, Peng X, Zhou M, Luo C, Nie Q, Zhang X. Polymorphisms of the IGF1R gene and their genetic effects on chicken early growth and carcass traits. BMC Genet. 2008;9:70 10.1186/1471-2156-9-70 . 18990245 PMC2628351

[33] Kaushal S, Schneider JW, Nadal-Ginard B, Mahdavi V. Activation of the myogenic lineage by MEF2A, a factor that induces and cooperates with MyoD. Science. 1994;266(5188):1236–40. . 7973707 10.1126/science.7973707

[34] Liu N, Nelson BR, Bezprozvannaya S, Shelton JM, Richardson JA, Bassel-Duby R, et al Requirement of MEF2A, C, and D for skeletal muscle regeneration. Proc Natl Acad Sci U S A. 2014;111(11):4109–14. 10.1073/pnas.1401732111 . 24591619 PMC3964114

[35] Shi W, Wymore R, Yu H, Wu J, Wymore RT, Pan Z, et al Distribution and prevalence of hyperpolarization-activated cation channel (HCN) mRNA expression in cardiac tissues. Circ Res. 1999;85(1):e1–6. . 10400919 10.1161/01.res.85.1.e1

[36] Zicha S, Fernandez-Velasco M, Lonardo G, L'Heureux N, Nattel S. Sinus node dysfunction and hyperpolarization-activated (HCN) channel subunit remodeling in a canine heart failure model. Cardiovasc Res. 2005;66(3):472–81. 10.1016/j.cardiores.2005.02.011 . 15914112

[37] Chandler NJ, Greener ID, Tellez JO, Inada S, Musa H, Molenaar P, et al Molecular architecture of the human sinus node: insights into the function of the cardiac pacemaker. Circulation. 2009;119(12):1562–75. 10.1161/CIRCULATIONAHA.108.804369 . 19289639

[38] Fenske S, Krause SC, Hassan SI, Becirovic E, Auer F, Bernard R, et al Sick sinus syndrome in HCN1-deficient mice. Circulation. 2013;128(24):2585–94. 10.1161/CIRCULATIONAHA.113.003712 . 24218458

[39] Van Laere AS, Nguyen M, Braunschweig M, Nezer C, Collette C, Moreau L, et al A regulatory mutation in IGF2 causes a major QTL effect on muscle growth in the pig. Nature. 2003;425(6960):832–6. 10.1038/nature02064 . 14574411

[40] Clark DL, Clark DI, Hogan EK, Kroscher KA, Dilger AC. Elevated insulin-like growth factor 2 expression may contribute to the hypermuscular phenotype of myostatin null mice. Growth Horm IGF Res. 2015;25(5):207–18. 10.1016/j.ghir.2015.06.007 . 26198127

[41] Farmer WT, Farin PW, Piedrahita JA, Bischoff SR, Farin CE. Expression of antisense of insulin-like growth factor-2 receptor RNA non-coding (AIRN) during early gestation in cattle. Anim Reprod Sci. 2013;138(1–2):64–73. 10.1016/j.anireprosci.2013.01.009 . 23473694

[42] Micke GC, Sullivan TM, McMillen IC, Gentili S, Perry VE. Protein intake during gestation affects postnatal bovine skeletal muscle growth and relative expression of IGF1, IGF1R, IGF2 and IGF2R. Mol Cell Endocrinol. 2011;332(1–2):234–41. 10.1016/j.mce.2010.10.018 . 21056085

[43] Marchler-Bauer A, Derbyshire MK, Gonzales NR, Lu S, Chitsaz F, Geer LY, et al CDD: NCBI's conserved domain database. Nucleic Acids Res. 2015;43(Database issue):D222–6. 10.1093/nar/gku1221 . 25414356 PMC4383992

[44] Morgan DO, Edman JC, Standring DN, Fried VA, Smith MC, Roth RA, et al Insulin-like growth factor II receptor as a multifunctional binding protein. Nature. 1987;329(6137):301–7. 10.1038/329301a0 . 2957598

[45] Devi GR, Byrd JC, Slentz DH, MacDonald RG. An insulin-like growth factor II (IGF-II) affinity-enhancing domain localized within extracytoplasmic repeat 13 of the IGF-II/mannose 6-phosphate receptor. Mol Endocrinol. 1998;12(11):1661–72. 10.1210/mend.12.11.0192 . 9817593

[46] Andersson L. Studying phenotypic evolution in domestic animals: a walk in the footsteps of Charles Darwin. Cold Spring Harb Symp Quant Biol. 2009;74:319–25. 10.1101/sqb.2009.74.039 . 20375320

[47] Andersson L. Detecting loci under selection using whole genome resequencing International Plant and Animal Genome Conference Xxi. San Diego, CA. USA, 2013.

[48] Wang L, Li X, Ma J, Zhang Y, Zhang H. Integrating genome and transcriptome profiling for elucidating the mechanism of muscle growth and lipid deposition in Pekin ducks. Sci Rep. 2017;7(1):3837 10.1038/s41598-017-04178-7 . 28630415 PMC5476626

[49] Wu Y, Zhang HL, Wang J, Liu XL. Discovery of a SNP in exon 7 of the lipoprotein lipase gene and its association with fatness traits in native and Cherry Valley Peking ducks. Anim Genet. 2008;39(5):564–6. 10.1111/j.1365-2052.2008.01761.x . 18671687

[50] Kurtz S, Phillippy A, Delcher AL, Smoot M, Shumway M, Antonescu C, et al Versatile and open software for comparing large genomes. Genome Biol. 2004;5(2):R12 10.1186/gb-2004-5-2-r12 . 14759262 PMC395750

[51] Xu TS, Gu LH, Sun Y, Zhang XH, Ye BG, Liu XL, et al Characterization of MUSTN1 gene and its relationship with skeletal muscle development at postnatal stages in Pekin ducks. Genet Mol Res. 2015;14(2):4448–60. 10.4238/2015.May.4.2 . 25966217

[52] Li H, Durbin R. Fast and accurate short read alignment with Burrows-Wheeler transform. Bioinformatics. 2009;25(14):1754–60. . 19451168 10.1093/bioinformatics/btp324 PMC2705234

[53] De Summa S, Malerba G, Pinto R, Mori A, Mijatovic V, Tommasi S. GATK hard filtering: tunable parameters to improve variant calling for next generation sequencing targeted gene panel data. BMC Bioinformatics. 2017;18(Suppl 5):119 10.1186/s12859-017-1537-8 . 28361668 PMC5374681

[54] Hansen NF. Variant calling from next generation sequence data. Methods Mol Biol. 2016;1418:209–24. 10.1007/978-1-4939-3578-9_11 . 27008017

[55] Cingolani P, Platts A, Wang le L, Coon M, Nguyen T, Wang L, et al A program for annotating and predicting the effects of single nucleotide polymorphisms, SnpEff: SNPs in the genome of Drosophila melanogaster strain w1118; iso-2; iso-3. Fly (Austin). 2012;6(2):80–92. 10.4161/fly.19695 . 22728672 PMC3679285

[56] Kofler R, Pandey RV, Schlotterer C. PoPoolation2: identifying differentiation between populations using sequencing of pooled DNA samples (Pool-Seq). Bioinformatics. 2011;27(24):3435–6. 10.1093/bioinformatics/btr589 . 22025480 PMC3232374

[57] Katoh K, Toh H. Recent developments in the MAFFT multiple sequence alignment program. Brief Bioinform. 2008;9(4):286–98. 10.1093/bib/bbn013 . 18372315

[58] Falcon S, Gentleman R. Using GOstats to test gene lists for GO term association. Bioinformatics. 2007;23(2):257–8. 10.1093/bioinformatics/btl567 . 17098774

[59] Papadopoulos JS, Agarwala R. COBALT: constraint-based alignment tool for multiple protein sequences. Bioinformatics. 2007;23(9):1073–9. 10.1093/bioinformatics/btm076 . 17332019

[60] Xu TS, Gu LH, Huang W, Xia WL, Zhang YS, Zhang YG, et al Gene expression profiling in Pekin duck embryonic breast muscle. PLoS One. 2017;12(5):e0174612 10.1371/journal.pone.0174612 . 28472139 PMC5417483

