# Correction: Analysis of *Anasplatyrhynchos* genome resequencing data reveals genetic signatures of artificial selection

**DOI:** 10.1371/journal.pone.0220996

**Published:** 2019-08-05

**Authors:** Tieshan Xu, Lihong Gu, Haopeng Yu, Xuefei Jiang, Yunsheng Zhang, Xiaohui Zhang, Guang Rong, Zhengkui Zhou, Kyle M. Schachtschneider, Shuisheng Hou

The word *Anasplatyrhynchos* appears incorrectly in the title, citation, and first sentence of the second paragraph of the Introduction section. The correct word is *Anas platyrhynchos*.

There are errors in the author affiliations. The correct affiliations are as follows: Tieshan Xu^1,2^, Lihong Gu^3^, Haopeng Yu^4^, Xuefei Jiang^5^, Yunsheng Zhang^2^, Xiaohui Zhang^6^, Guang Rong^1^, Zhengkui Zhou^2^, Kyle M. Schachtschneider^7^, Shuisheng Hou^2^

**1** Tropical Crop Genetic Resource Research Institute, Chinese Academy of Tropical Agricultural Sciences, Danzhou, P.R. China, **2** Institute of Animal Science, Chinese Academy of Agricultural Sciences, Beijing, P.R. China, **3** Institute of Animal Science & Veterinary, Hainan Academy of Agricultural Science, Haikou, P.R. China, **4** West China Biomedical Big Data Center, West China Hospital/West China School of Medicine, Sichuan University, Chengdu, P.R. China, **5** Institute of Tropical Agriculture and Forestry, Hainan University, Haikou, P.R. China, **6** College of Animal Science, Henan University of Science and Technology, Luoyang P. R. China, **7** Department of Radiology, University of Illinois at Chicago, Chicago, Illinois, United States of America
