# Supplementary figures and images for: Analysis of Anasplatyrhynchos genome resequencing data reveals genetic signatures of artificial selection
Source: PLoS One. 2019 Feb 8;14(2):e0211908. doi: 10.1371/journal.pone.0211908 (PMC6368380; doi:10.1371/journal.pone.0211908)

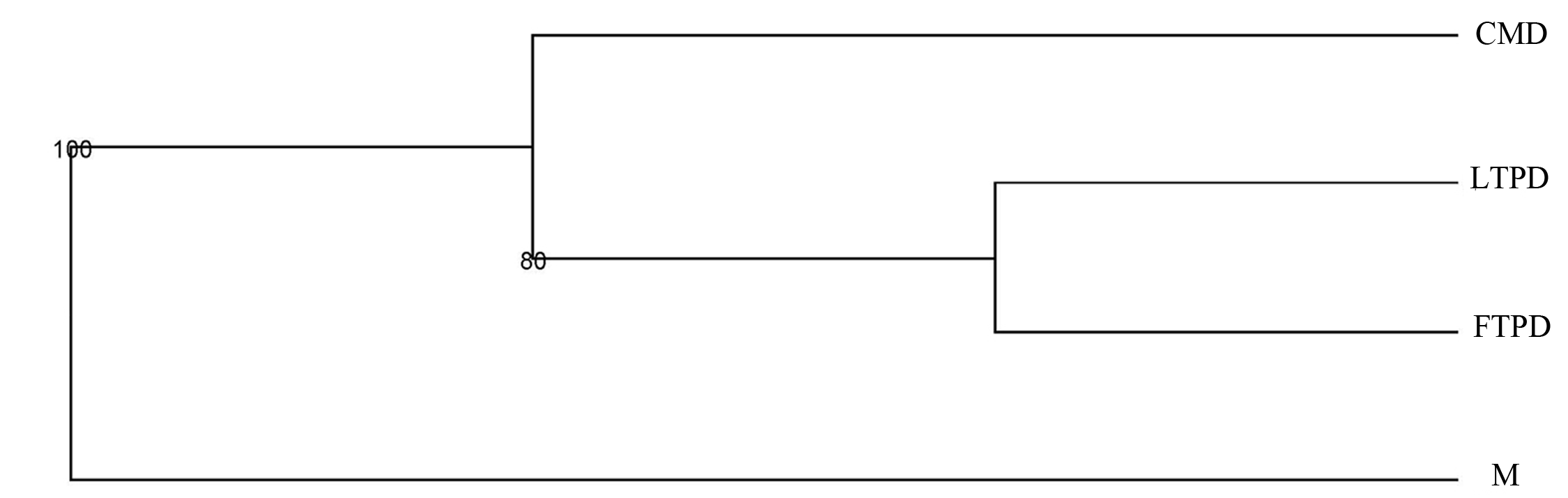

Supplement: S1 Fig — (TIF) [file pone.0211908.s001.tif]

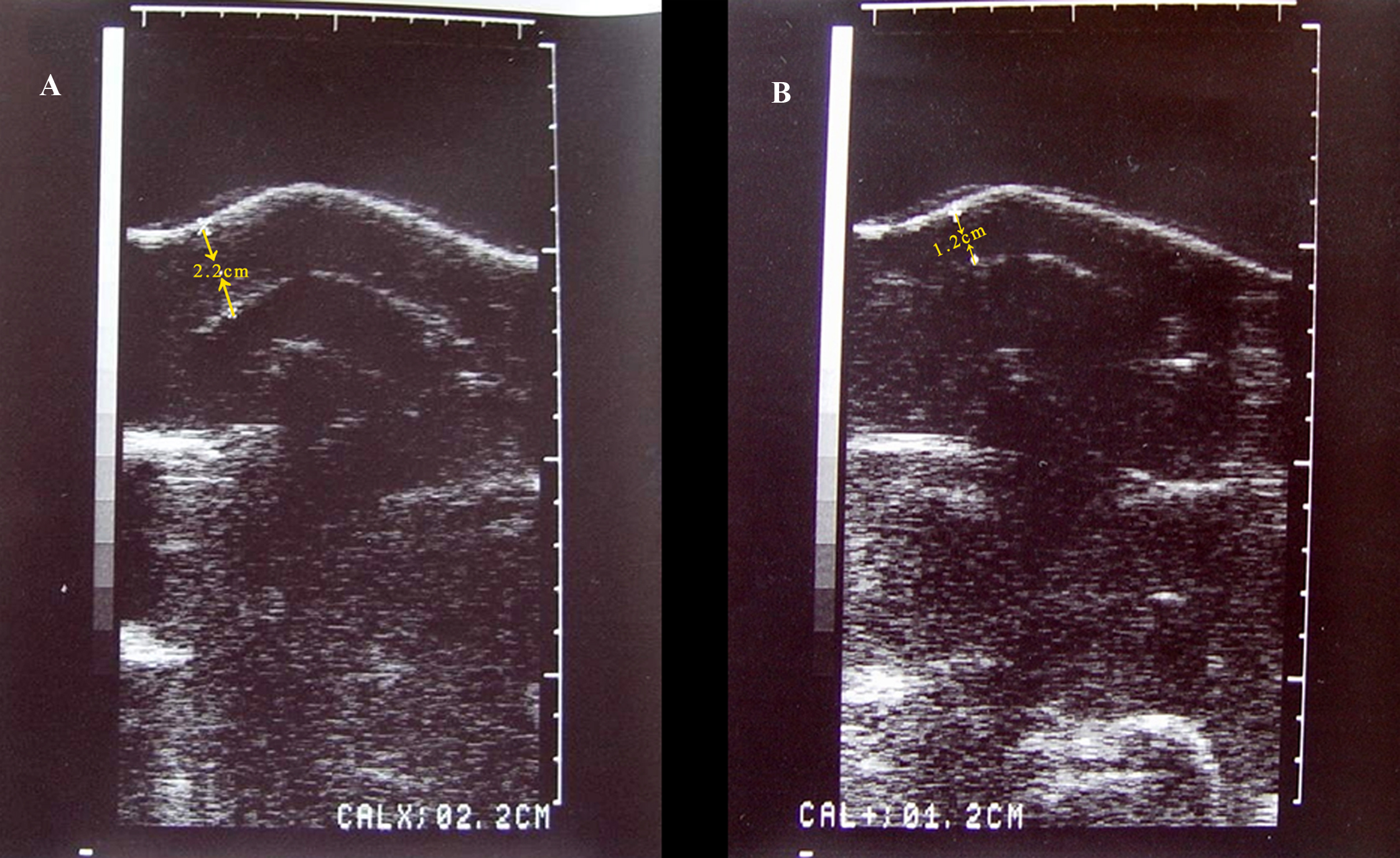

Supplement: S2 Fig — a, The breast muscle thickness of LTPD. b, The breast muscle thickness of FTPD. (TIF) [file pone.0211908.s002.tif]

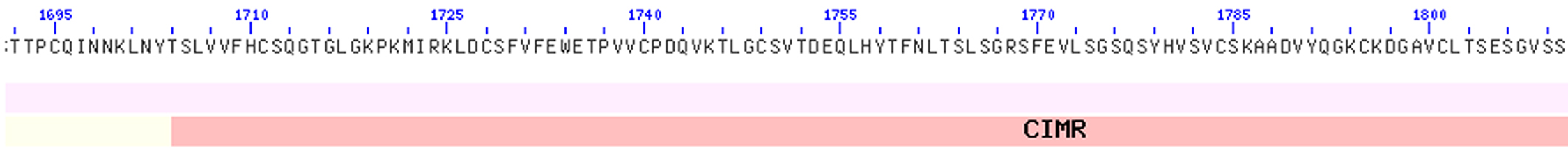

Supplement: S3 Fig — (TIF) [file pone.0211908.s003.tif]

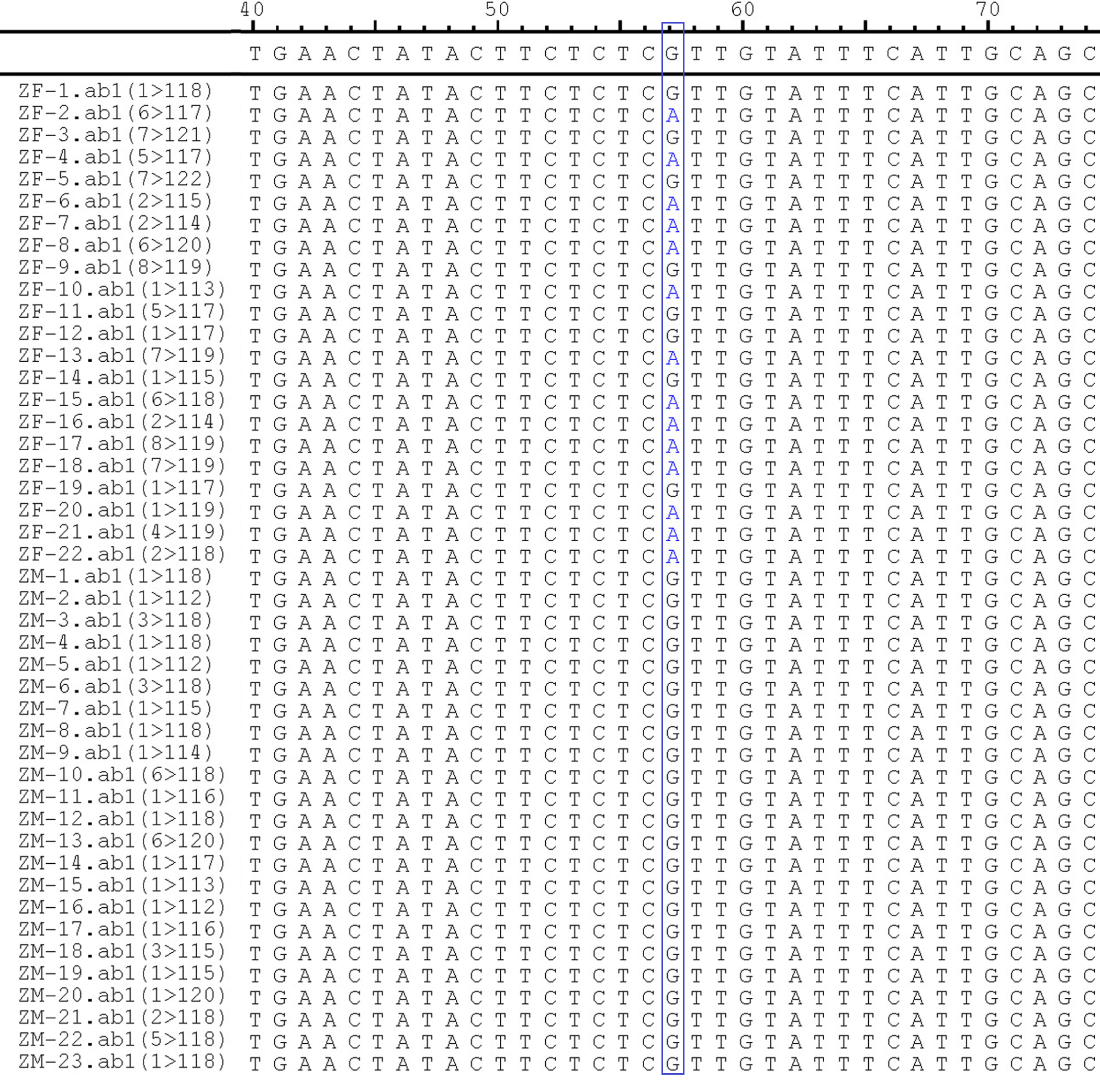

Supplement: S4 Fig — All of “A” at this site were marked in blue. (TIF) [file pone.0211908.s004.tif]

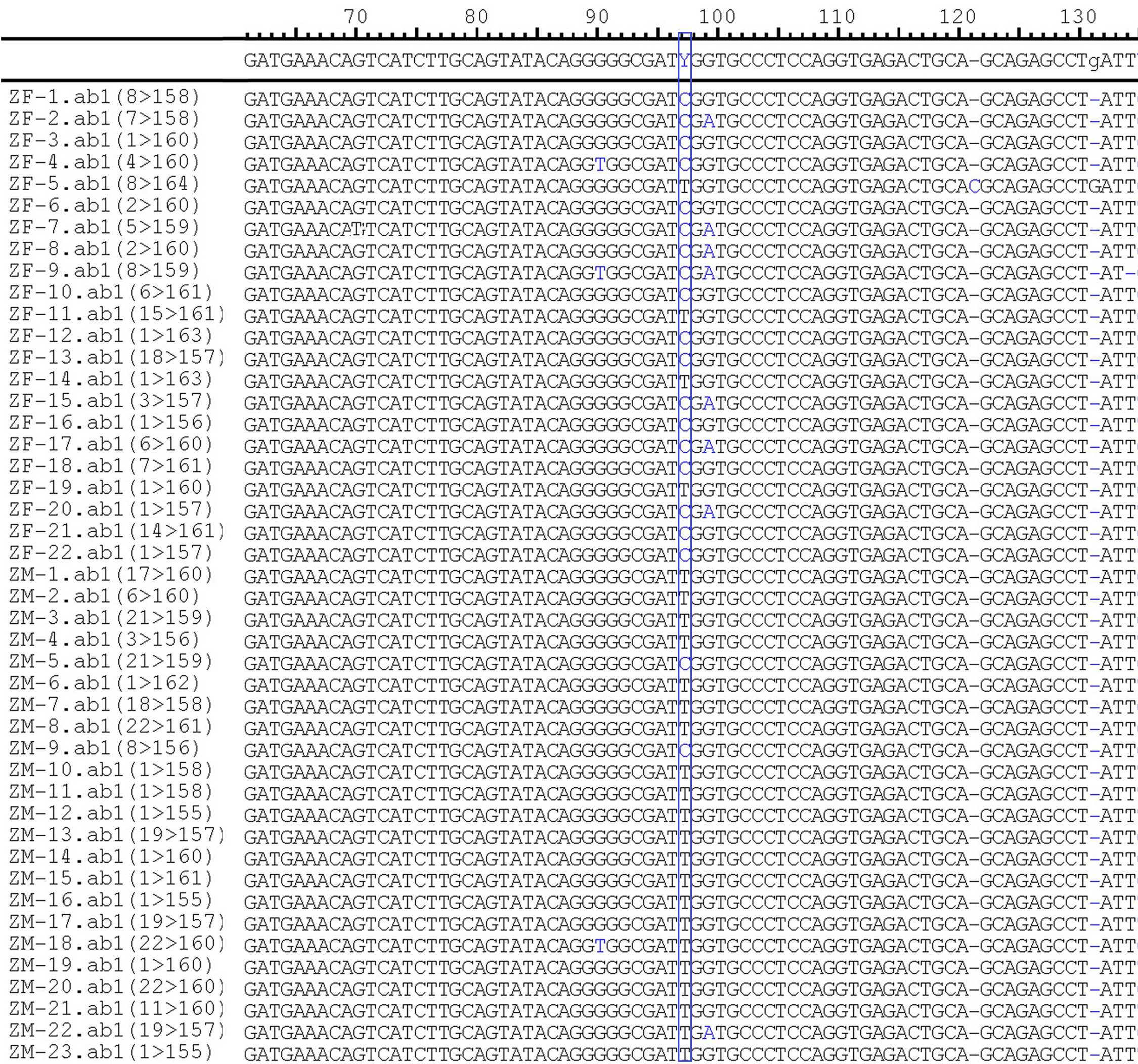

Supplement: S5 Fig — All of “C” at this site were marked in blue. (TIF) [file pone.0211908.s005.tif]

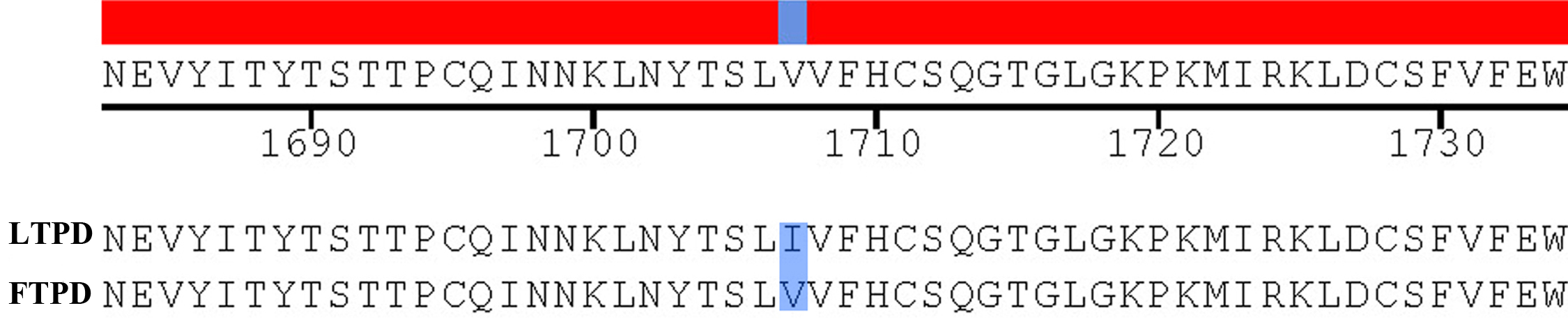

Supplement: S6 Fig — “V” and “I” at this site were marked in blue. (TIF) [file pone.0211908.s006.tif]

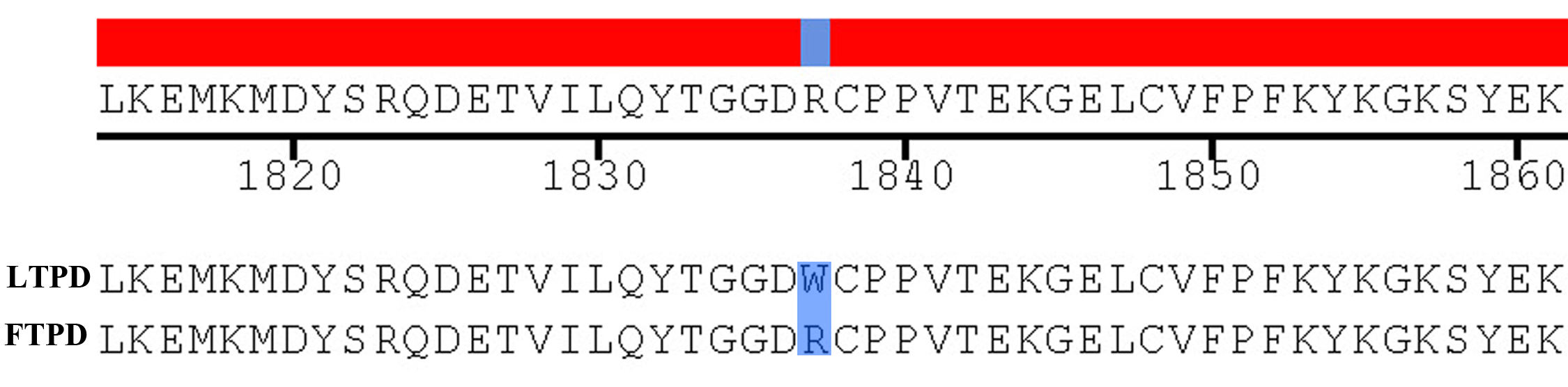

Supplement: S7 Fig — “W” and “R” at this site were marked in blue. (TIF) [file pone.0211908.s007.tif]
