# Supplementary material for: Analysis of Anasplatyrhynchos genome resequencing data reveals genetic signatures of artificial selection
Source: PLoS One. 2019 Feb 8;14(2):e0211908. doi: 10.1371/journal.pone.0211908 (PMC6368380; doi:10.1371/journal.pone.0211908)
Supplement: S1 Table — (DOCX) [file pone.0211908.s008.docx]

**S1 Table. Comparison of four duck populations studied in this paper** (Values are means ± s.d).

| Items/Species | Fat-type Pekin ducks (FTPD) | Lean-type Pekin ducks (LTPD) | China Micro-Duck (CMD) | Mallard (M) |
| --- | --- | --- | --- | --- |
| Appearance | 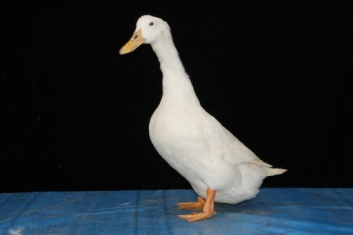 | 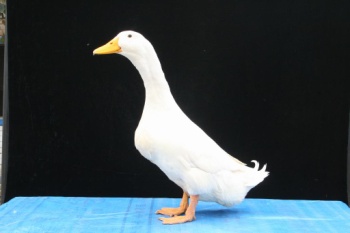 | 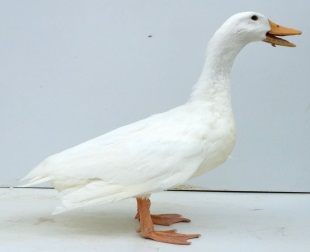 | 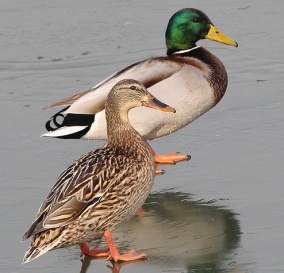 |
| Breed history | The breed originated in north China, mainly in Beijing region. The ZF line has been intensively artificially selected for fast growth and high fat rate. Conversely, the ZM line has been intensively artificially selected mainly for high lean meat rate with the considering of growth performance. | | Indigenous breed in south China that was collected by Institute of Animal Science, CAAS, China for white feather and compact body shape. | This breed has been artificially selected to some degree for egg and meat production. |
| Breed purpose | Providing raw materials of Beijing roast duck | Providing raw material for Chinese meat duck market | To meet the need of making high quality duck meat products. | Providing high quality eggs. |
| Characteristics | White feather.  Large body size and fast growth. The average body weight at 6 weeks of age is 3206±217.9 g (n=1953). The average daily gain from birth to 42 days is 75±18.9 g (n=1953).  High fat percentage and low lean meat percentage. The average fat (skin fat + abdomen fat) percentage is 32.23±2.16% (male is 32.47% and female is 32.00%), and the average lean meat (breast muscle + leg muscle) percentage is 20.96±1.83% (male is 21.22% and female is 20.70%). | White feather.  Large body size and fast growth. The average body weight at 6 weeks of age is 3018±223.4 g (n=2350) g. The average daily gain from birth to 42 days is 72±17.8 g (n=2350).  High lean meat percentage and low fat percentage. The average lean meat (breast muscle + leg muscle) percentage is 26.13±1.95% (male is 26.32% and female is 25.93%), and the average fat (skin fat + abdomen fat) percentage is 25.00±1.99% (male is 24.09% and female is 26.08%). | White feather.  Small body size and slow growth. The average body weight at 65 days of age is 1795±112.5 g (n=1329) g. The average daily gain from birth to 65 days is 27±7.6 g (n=1329).  Medium lean meat percentage and fat percentage. The average lean meat (breast muscle + leg muscle) percentage is 23.83±1.77% (male is 23.70% and female is 23.95%), and the average fat (skin fat + abdomen fat) percentage is 25.85±2.03% (male is 25.35% and female is 26.35%). | Brown feathers.  Small body size and slow growth.  The average body weight at 100 days of age is 10725±68.2 g (n=839) g.  High lean meat percentage and low fat percentage. The average lean meat (breast muscle + leg muscle) percentage is 32.13±2.01% (n=256). |
| Reproduction | The average egg production at 66 weeks of age is 217±28.37, and the hatchability of hatched eggs is 89±2.24%. | The average egg production at 66 weeks of age is 223±27.96, and the hatchability of hatched eggs is 92±2.31%. | Reproductive performance is excellent. The average egg production at 66 weeks of age is 289.38±22.16, and the hatchability of fertilized eggs is 94.47±3.59%. | Reproductive performance is poor. The average egg production per year is 62.86±17.25, and the hatchability of fertilized eggs is 85%~90%. |
| Current distribution | Major in Beijing city and its  surrounding areas | Major in Beijing city and its  surrounding areas | The breeding farm of Z-type Pekin ducks of Institute of Animal Sciences, CAAS. | Distributed in the temperate waters of northern part of Europe, Asia and the Americas. |
