# Supplementary material for: Analysis of Anasplatyrhynchos genome resequencing data reveals genetic signatures of artificial selection
Source: PLoS One. 2019 Feb 8;14(2):e0211908. doi: 10.1371/journal.pone.0211908 (PMC6368380; doi:10.1371/journal.pone.0211908)
Supplement: S2 Table — (DOCX) [file pone.0211908.s009.docx]

**S2 Table. The results of anchoring duck scaffolds to psuo-chromosomes**

| Psuo-chromosomes | Contained scaffold IDs | Queried length (bps) |
| --- | --- | --- |
| 1 | KB742830.1, KB742566.1, KB742427.1, KB742722.1, KB747348.1, KB743833.1, KB742662.1, KB743338.1, KB742435.1, KB743037.1, KB745568.1, KB742499.1, KB742798.1, KB743360.1, KB742864.1, KB742675.1, KB743733.1, KB742471.1, KB743204.1, KB743534.1, KB742648.1, KB742646.1, KB743807.1, KB743637.1, KB742515.1, KB742647.1, KB743050.1, KB742939.1, KB742451.1, KB742520.1, KB743213.1, KB744799.1, KB743692.1, KB744042.1, KB742384.1, KB743608.1, KB743303.1, KB743519.1, KB742497.1, KB744222.1, KB744795.1, KB742993.1, KB743700.1, KB742595.1, KB743162.1, KB743625.1, KB742585.1, KB744454.1, KB743199.1, KB744797.1, KB744942.1, KB744410.1, KB742823.1, KB742784.1, KB742550.1, KB743490.1, KB743107.1, KB744504.1, KB743378.1, KB744863.1, KB743260.1, KB743151.1, KB743476.1, KB745754.1, KB743919.1, KB742547.1, KB742468.1, KB743194.1, KB742449.1, KB742887.1, KB743435.1, KB743948.1, KB744900.1, KB745238.1, KB744673.1, KB743149.1, KB742459.1, KB742556.1, KB742597.1, KB745111.1, KB744509.1, KB744434.1, KB744551.1, KB742870.1, KB742847.1, KB743949.1, KB743297.1, KB742756.1, KB743339.1, KB743424.1, KB742906.1, KB743167.1, KB745072.1, KB743866.1, KB744839.1, KB742463.1, KB744027.1, KB742440.1, KB743004.1, KB742942.1, KB743564.1, KB744640.1, KB742943.1, KB742931.1, KB743316.1, KB743899.1, KB745404.1, KB743292.1, KB743121.1, KB742543.1, KB743441.1, KB742951.1, KB742682.1, KB742627.1, KB742411.1, KB743889.1, KB745737.1, KB743101.1, KB743059.1, KB742605.1, KB742710.1, KB742858.1, KB743351.1, KB742496.1, KB742504.1, KB744451.1, KB742701.1, KB742774.1, KB744694.1, KB742810.1, KB742570.1, KB742469.1, KB743751.1, KB743240.1, KB743662.1, KB744657.1, KB742659.1, KB747347.1, KB742598.1, KB745428.1, KB743208.1, KB743304.1, KB743295.1, KB742397.1, KB747282.1, KB744180.1, KB743282.1, KB743137.1, KB742744.1, KB743154.1, KB744237.1, KB745115.1, KB744494.1, KB742739.1, KB742999.1, KB742439.1, KB742423.1, KB743006.1, KB743300.1, KB742645.1, KB742866.1, KB743501.1, KB743032.1, KB742389.1, KB745334.1, KB744475.1, KB743653.1, KB743475.1, KB742778.1, KB744073.1, KB743630.1, KB742740.1, KB745040.1, KB742992.1, KB742680.1, KB742973.1, KB742923.1, KB743893.1, KB743380.1, KB749111.1, KB744528.1, KB742725.1, KB743885.1, KB742842.1, KB742481.1, KB742622.1, KB744860.1, KB743568.1, KB744255.1, KB743904.1, KB743192.1, KB744949.1, KB743590.1, KB743562.1, KB743381.1, KB743796.1, KB744216.1 | 171,467,884 |
| 2 | KB744625.1, KB743777.1, KB742599.1, KB743458.1, KB743530.1, KB743471.1, KB745644.1, KB744154.1, KB744366.1, KB743054.1, KB743973.1, KB743531.1, KB744924.1, KB743896.1, KB742395.1, KB742804.1, KB742539.1, KB745104.1, KB744292.1, KB742630.1, KB743574.1, KB742631.1, KB742442.1, KB746063.1, KB744727.1, KB745567.1, KB744175.1, KB742650.1, KB742771.1, KB742957.1, KB742513.1, KB742964.1, KB742773.1, KB743177.1, KB743583.1, KB743319.1, KB743116.1, KB744844.1, KB743865.1, KB745141.1, KB744012.1, KB744720.1, KB745793.1, KB742966.1, KB744099.1, KB743611.1, KB743773.1, KB743326.1, KB745267.1, KB742856.1, KB742911.1, KB745003.1, KB742718.1, KB743065.1, KB743391.1, KB743537.1, KB742702.1, KB742690.1, KB742750.1, KB742535.1, KB742720.1, KB742991.1, KB743230.1, KB743237.1, KB743289.1, KB742775.1, KB742735.1, KB743529.1, KB745301.1, KB742889.1, KB744348.1, KB743436.1, KB743575.1, KB742877.1, KB742490.1, KB743404.1, KB743636.1, KB744519.1, KB743220.1, KB742836.1, KB743413.1, KB742844.1, KB743913.1, KB743678.1, KB744749.1, KB743400.1, KB743821.1, KB743886.1, KB743685.1, KB748326.1, KB745541.1, KB742486.1, KB742691.1, KB744243.1, KB744601.1, KB743438.1, KB744256.1, KB743579.1, KB742567.1, KB743633.1, KB742975.1, KB744214.1, KB742642.1, KB742524.1, KB742418.1, KB742424.1, KB742981.1, KB742466.1, KB742667.1, KB743786.1, KB745336.1, KB743251.1, KB744197.1, KB742582.1, KB742618.1, KB742428.1, KB742732.1, KB743076.1, KB743235.1, KB744276.1, KB743956.1, KB742824.1, KB743094.1, KB743635.1, KB742846.1, KB742403.1, KB742867.1, KB744834.1, KB743031.1, KB745269.1, KB742405.1, KB744088.1, KB742485.1, KB743875.1, KB743421.1, KB743132.1, KB742649.1, KB742412.1, KB742707.1, KB742457.1, KB742933.1, KB743558.1, KB743053.1, KB746368.1, KB743178.1, KB742977.1, KB742483.1, KB745085.1, KB742979.1, KB742862.1, KB742828.1, KB743277.1, KB744403.1 | 135,995,573 |
| 3 | KB743040.1, KB743395.1, KB743457.1, KB743111.1, KB742580.1, KB743307.1, KB742970.1, KB743722.1, KB743374.1, KB742503.1, KB742875.1, KB742890.1, KB743318.1, KB742799.1, KB743872.1, KB742430.1, KB742734.1, KB744474.1, KB742781.1, KB742444.1, KB742871.1, KB743511.1, KB745002.1, KB742809.1, KB743545.1, KB745424.1, KB743878.1, KB743336.1, KB743298.1, KB744936.1, KB742963.1, KB743520.1, KB743543.1, KB743832.1, KB747043.1, KB743594.1, KB743153.1, KB742905.1, KB745911.1, KB743070.1, KB743670.1, KB742815.1, KB743261.1, KB742386.1, KB743651.1, KB744251.1, KB742478.1, KB742980.1, KB743818.1, KB742760.1, KB744185.1, KB742456.1, KB742937.1, KB745610.1, KB742694.1, KB742432.1, KB742912.1, KB742860.1, KB743729.1, KB742416.1, KB745283.1, KB743494.1, KB742848.1, KB744089.1, KB743546.1, KB742658.1, KB743252.1, KB742594.1, KB743808.1, KB744604.1, KB742639.1, KB744050.1, KB743278.1, KB743735.1, KB742953.1, KB744588.1, KB742441.1, KB743200.1, KB743086.1, KB742406.1, KB742854.1, KB742592.1, KB742611.1, KB743007.1 , KB742699.1, KB742696.1, KB742484.1, KB742764.1, KB742629.1, KB742922.1, KB745622.1, KB743999.1, KB743997.1, KB747499.1, KB746281.1, KB747284.1, KB743561.1, KB743569.1, KB745020.1, KB744771.1, KB746044.1, KB744359.1, KB742693.1, KB745661.1 | 111,884,621 |
| 4 | KB743210.1, KB743046.1, KB743099.1, KB743186.1, KB742855.1, KB748974.1, KB743294.1, KB743532.1, KB742904.1, KB742549.1, KB742437.1, KB742574.1, KB743393.1, KB742393.1, KB743527.1, KB742777.1, KB743323.1, KB743176.1, KB743211.1, KB742853.1, KB744921.1, KB743013.1, KB742433.1, KB744106.1, KB743357.1, KB742733.1, KB743100.1, KB743113.1, KB744297.1, KB744927.1, KB745806.1, KB742590.1, KB743614.1, KB744195.1, KB744295.1, KB742619.1, KB748377.1, KB743718.1, KB744596.1, KB744150.1, KB743302.1, KB753339.1, KB742565.1, KB742801.1, KB742664.1, KB743632.1, KB743012.1, KB743609.1, KB744145.1, KB742932.1, KB742391.1, KB743713.1, KB742678.1, KB744333.1, KB742816.1, KB742793.1, KB746632.1, KB746855.1, KB744909.1, KB742949.1, KB743126.1, KB743072.1, KB820867.1, KB742948.1, KB742628.1, KB742878.1, KB742898.1, KB744293.1, KB743090.1, KB743358.1, KB742833.1, KB743657.1, KB745022.1, KB743464.1, KB742464.1, KB743555.1, KB742644.1, KB743566.1 | 85,984,428 |
| 5 | KB743325.1, KB744755.1, KB744634.1, KB743305.1, KB743140.1, KB745096.1, KB743062.1, KB744758.1, KB745938.1, KB745312.1, KB742752.1, KB743434.1, KB744094.1, KB743317.1, KB743019.1, KB744206.1, KB742934.1, KB742745.1, KB743645.1, KB744679.1, KB743214.1, KB742766.1, KB744435.1, KB743616.1, KB744162.1, KB742884.1, KB743581.1, KB743367.1, KB743402.1, KB742537.1, KB742623.1, KB744445.1, KB743578.1, KB743071.1, KB745135.1, KB744512.1, KB742689.1, KB747070.1, KB744676.1, KB745717.1, KB743275.1, KB743356.1, KB742443.1, KB743183.1, KB743383.1,. KB742730.1, KB743256.1, KB742685.1, KB742946.1, KB744486.1, KB745378.1, KB743257.1, KB743197.1, KB743450.1, KB745397.1, KB745110.1, KB743650.1, KB743804.1, KB743396.1, KB744739.1, KB745078.1, KB744248.1, KB744710.1, KB742996.1, KB743205.1, KB742861.1, KB743675.1, KB742921.1, KB744026.1, KB743721.1 | 53,360,204 |
| 6 | KB743944.1 KB742802.1 KB744430.1 KB742808.1 KB743728.1  KB743335.1 KB744772.1 KB742671.1 KB742660.1 KB744431.1  KB743484.1 KB742476.1 KB743454.1 KB742512.1 KB744554.1  KB743871.1 KB746205.1 KB744008.1 KB742388.1 KB744674.1  KB742714.1 KB743308.1 KB743226.1 KB743453.1 KB743726.1  KB743340.1 KB742609.1 KB744933.1 KB743109.1 | 31,073,993 |
| 7 | KB742542.1 KB743901.1 KB746286.1 KB743870.1 KB743291.1  KB744105.1 KB743930.1 KB744196.1 KB743414.1 KB743366.1  KB743649.1 KB743811.1 KB744270.1 KB744466.1 KB742719.1  KB742873.1 KB742668.1 KB743108.1 KB743045.1 KB744072.1  KB745786.1 KB743225.1 KB749062.1 KB742511.1 KB745880.1  KB742929.1 KB745056.1 KB743158.1 KB742571.1 KB743688.1  KB742462.1 KB742819.1 KB744209.1 | 35,065,787 |
| 8 | KB744465.1 KB744681.1 KB744156.1 KB743680.1 KB742477.1  KB744174.1 KB742383.1 KB743747.1 KB744349.1 KB743906.1  KB743073.1 KB744638.1 KB744429.1 KB743021.1 KB744110.1  KB744020.1 KB744722.1 KB743342.1 KB742473.1 KB742408.1  KB744547.1 KB743274.1 KB743203.1 KB742606.1 KB744170.1  KB744212.1 KB744148.1 KB744139.1 KB743201.1 KB743682.1  KB743412.1 KB749012.1 KB743788.1 KB744328.1 KB743348.1  KB743069.1 KB747321.1 | 27,610,499 |
| 9 | KB743681.1 KB744955.1 KB744631.1 KB742711.1 KB743085.1  KB744332.1 KB744591.1 KB744477.1 KB743330.1 KB743977.1  KB743629.1 KB743780.1 KB742505.1 KB743410.1 KB744899.1  KB746984.1 KB744039.1 KB743359.1 KB743644.1 KB742924.1  KB743103.1 KB743198.1 KB751064.1 KB744208.1 KB742612.1  KB743975.1 KB743525.1 | 22,719,958 |
| 10 | KB743447.1 KB744986.1 KB743248.1 KB743455.1 KB743702.1  KB744306.1 KB743771.1 KB742572.1 KB743900.1 KB746221.1  KB746118.1 KB744125.1 KB743097.1 KB744077.1 KB744113.1  KB746217.1 KB745158.1 KB744224.1 KB742888.1 KB742523.1  KB743789.1 KB742800.1 KB745236.1 KB743431.1 KB753939.1  KB744444.1 KB742410.1 KB743161.1 KB743783.1 | 19,578,582 |
| 11 | KB744937.1 KB742706.1 KB743822.1 KB746224.1 KB742399.1  KB743829.1 KB742880.1 KB743273.1 KB746316.1 KB743571.1  KB743565.1 KB743157.1 KB743139.1 KB742984.1 KB743146.1  KB748162.1 KB742851.1 KB742958.1 KB742421.1 KB744525.1  KB742947.1 KB745790.1 KB743859.1 KB744558.1 | 20,012,626 |
| 12 | KB743740.1 KB747245.1 KB744000.1 KB744093.1 KB744329.1  KB743544.1 KB743209.1 KB744234.1 KB743814.1 KB742679.1  KB742540.1 KB742849.1 KB743058.1 KB743962.1 KB743551.1  KB742569.1 KB743709.1 KB743891.1 KB742915.1 KB744074.1  KB742541.1 KB743459.1 KB742789.1 KB742527.1 KB743828.1  KB744362.1 KB743136.1 KB742579.1 | 21,069,769 |
| 13 | KB742509.1 KB742452.1 KB743686.1 KB743509.1 KB744621.1  KB743041.1 KB742414.1 KB744553.1 KB746070.1 KB744079.1  KB745225.1 KB744344.1 KB742965.1 KB744300.1 KB742795.1  KB745046.1 KB743924.1 KB742482.1 KB744917.1 KB743585.1  KB743857.1 KB744501.1 KB743017.1 KB742531.1 KB746227.1  KB743600.1 KB743369.1 KB743364.1 | 19,476,806 |
| 14 | KB744498.1 KB743145.1 KB742987.1 KB743091.1 KB742528.1  KB742883.1 KB742553.1 KB744307.1 KB748070.1 KB743477.1  KB744706.1 KB744586.1 KB744448.1 KB743009.1 KB743005.1  KB744198.1 KB742404.1 KB744458.1 KB742700.1 KB744666.1  KB743321.1 KB742394.1 KB743068.1 KB744413.1 KB742454.1  KB743669.1 | 17,143,714 |
| 15 | KB743228.1 KB744734.1 KB743603.1 KB742969.1 KB742438.1  KB742840.1 KB744353.1 KB744610.1 KB745522.1 KB742615.1  KB743965.1 KB743809.1 KB743423.1 KB742723.1 KB744908.1  KB745285.1 KB743518.1 KB744033.1 KB745183.1 KB743972.1  KB743627.1 KB744108.1 KB742807.1 | 14,131,511 |
| 17 | KB742736.1 KB743350.1 KB744204.1 KB744347.1 KB743352.1  KB745035.1 KB743815.1 KB742955.1 KB742651.1 KB742770.1  KB743399.1 KB743801.1 KB745179.1 KB744822.1 KB743610.1  KB743556.1 | 11,098,027 |
| 18 | KB742724.1 KB745034.1 KB742686.1 KB744582.1 KB746093.1  KB743855.1 KB744059.1 KB742959.1 KB743105.1 KB742703.1  KB742465.1 KB742826.1 KB742588.1 KB742832.1 KB743912.1  KB744567.1 KB743370.1 KB743018.1 KB745223.1 KB747351.1  KB743008.1 KB744629.1 KB742551.1 | 10,518,134 |
| 19 | KB742811.1 KB744562.1 KB742480.1 KB742940.1 KB744160.1  KB742489.1 KB744607.1 KB743696.1 KB743446.1 KB742712.1  KB742616.1 KB743217.1 | 11,556,554 |
| 20 | KB742967.1 KB743057.1 KB742559.1 KB743547.1 KB742845.1  KB744800.1 KB744646.1 KB742407.1 KB744068.1 KB744325.1  KB743768.1 KB742920.1 KB742521.1 KB745027.1 KB743541.1  KB743881.1 KB742704.1 KB746278.1 | 8,810,297 |
| 21 | KB742522.1 KB742382.1 KB745036.1 KB743580.1 KB742794.1  KB743963.1 KB742757.1 KB743246.1 KB742716.1 KB744266.1 | 7,739,914 |
| 22 | KB743612.1 KB743507.1 KB744520.1 KB743642.1 KB743926.1  KB745279.1 KB742665.1 KB744812.1 KB742995.1 | 3,314,160 |
| 23 | KB742797.1 KB743739.1 KB744656.1 KB743420.1 KB743479.1  KB744345.1 KB744461.1 KB743174.1 KB744367.1 KB743745.1  KB743762.1 KB743749.1 KB743388.1 KB743175.1 KB742637.1 | 6,708,138 |
| 24 | KB743221.1 KB743106.1 KB743638.1 KB742534.1 KB744031.1  KB743456.1 KB744376.1 KB744100.1 KB745640.1 KB743619.1  KB742431.1 KB743227.1 KB746795.1 | 6,949,570 |
| 25 | KB745294.1 KB743191.1 KB743403.1 KB748399.1 KB744049.1  KB743560.1 | 971,270 |
| 26 | KB747363.1 KB742726.1 KB742677.1 KB745055.1 KB743553.1  KB744902.1 KB742563.1 KB742896.1 KB743473.1 KB743510.1  KB744057.1 KB743493.1 | 6,077,055 |
| 27 | KB744289.1 KB743001.1 KB743937.1 KB743284.1 KB743408.1  KB746071.1 KB744725.1 KB743180.1 KB743922.1 KB744358.1  KB747705.1 KB744807.1 KB745531.1 KB744753.1 KB744132.1  KB742576.1 KB742525.1 | 3,763,040 |
| 28 | KB743016.1 KB744134.1 KB743098.1 KB744699.1 KB743311.1  KB744221.1 KB748146.1 KB745442.1 KB744754.1 KB743437.1  KB743647.1 KB743923.1 KB744974.1 KB744335.1 KB744667.1  KB744616.1 KB744239.1 | 3,819,860 |
| Z | KB742564.1 KB746388.1 KB743741.1 KB743487.1 KB743549.1  KB743344.1 KB742625.1 KB743206.1 KB742561.1 KB743599.1  KB744001.1 KB744263.1 KB743432.1 KB743089.1 KB747281.1  KB745723.1 KB743486.1 KB742544.1 KB743964.1 KB742895.1  KB743584.1 KB742613.1 KB742602.1 KB744087.1 KB742742.1  KB744159.1 KB743778.1 KB743120.1 KB743038.1 KB742983.1  KB742387.1 KB742632.1 KB742448.1 KB743658.1 KB744639.1  KB742554.1 KB743466.1 KB742640.1 KB742786.1 KB742741.1  KB743030.1 KB742985.1 KB742918.1 KB742813.1 KB743159.1  KB742655.1 KB742634.1 KB742850.1 KB742573.1 KB743241.1  KB742900.1 KB743152.1 KB743143.1 KB742834.1 KB743655.1  KB743087.1 KB743601.1 KB743958.1 KB742782.1 KB742583.1  KB743572.1 KB744904.1 KB744240.1 KB743288.1 KB743048.1  KB742652.1 KB744294.1 KB742806.1 KB742960.1 KB742935.1  KB743499.1 KB742491.1 KB743051.1 KB742791.1 KB743171.1  KB742746.1 KB746935.1 KB742876.1 KB751155.1 KB743868.1  KB743024.1 KB743474.1 KB742787.1 KB743026.1 KB742643.1  KB743957.1 KB743341.1 | 55,310,714 |
| Total | 1141 | 923,212,688 |
