# Supplementary material for: Analysis of Anasplatyrhynchos genome resequencing data reveals genetic signatures of artificial selection
Source: PLoS One. 2019 Feb 8;14(2):e0211908. doi: 10.1371/journal.pone.0211908 (PMC6368380; doi:10.1371/journal.pone.0211908)
Supplement: S3 Table — (DOCX) [file pone.0211908.s010.docx]

**S3 Table. Pool information for resequencing data from four duck populations.**

| Species | n | Sequence cov. |
| --- | --- | --- |
| FTPD | 30 | ~40× |
| LTPD | 30 | ~40× |
| CDM | 30 | ~40× |
| M | 30 | ~40× |
| Total | 120 | ~160× |
