# Supplementary material for: Analysis of Anasplatyrhynchos genome resequencing data reveals genetic signatures of artificial selection
Source: PLoS One. 2019 Feb 8;14(2):e0211908. doi: 10.1371/journal.pone.0211908 (PMC6368380; doi:10.1371/journal.pone.0211908)
Supplement: S4 Table — (DOCX) [file pone.0211908.s011.docx]

**S4 Table. Basic sequenced data statistics for the four duck population**

| Sample | Read pair number | Total_bases (Gb) | GC_content | Q20 | Q30 | Coverage(X) |
| --- | --- | --- | --- | --- | --- | --- |
| FTPD | 219,905,659 | 43.98 | 40.94% | 95.01% | 90.64% | 39.80 |
| LTPD | 228,789,774 | 51.76 | 41.55% | 93.17% | 87.67% | 46.84 |
| CMD | 224,920,852 | 44.98 | 41.42% | 95.24% | 90.67% | 40.71 |
| M | 226,178,409 | 45.24 | 41.50% | 94.68% | 89.19% | 40.94 |
| Total |  | 185.96 |  |  |  | 168.44 |
