# Supplementary material for: Analysis of Anasplatyrhynchos genome resequencing data reveals genetic signatures of artificial selection
Source: PLoS One. 2019 Feb 8;14(2):e0211908. doi: 10.1371/journal.pone.0211908 (PMC6368380; doi:10.1371/journal.pone.0211908)
Supplement: S5 Table — (DOCX) [file pone.0211908.s012.docx]

**S5 Table. Identified SNPs for each duck population**

| Mutation types | | FTPD | LTPD | CMD | M | All | |
| --- | --- | --- | --- | --- | --- | --- | --- |
|  |  |  |  |  |  | Count | % |
| Classification by location | 5’ UTR | 1,636 | 1,689 | 1,715 | 2,127 | 2,366 | 0.06 |
|  | 3’ UTR | 14,227 | 14,600 | 15,127 | 18,519 | 20,610 | 0.49 |
|  | Exon | 130,260 | 134,351 | 138,962 | 169,973 | 188,352 | 4.50 |
|  | Intron | 2,738,519 | 2,815,641 | 2,915,784 | 3,625,737 | 3,977,357 | 94.95 |
| Classification by impact | High | 1,027 | 1,040 | 1,087 | 1,302 | 1,431 | 0.01 |
|  | Low | 116,712 | 120,354 | 124,666 | 153,528 | 168,937 | 1.25 |
|  | Moderate | 28,382 | 29,218 | 30,080 | 35,820 | 40,642 | 0.30 |
|  | Modifier | 9,144,361 | 9,405,165 | 9,740,647 | 12,100,753 | 13,350,965 | 98.44 |
| Classification by functional class | Missense | 28,401 | 29,238 | 30,104 | 35,850 | 40,676 | 21.28 |
|  | Nonsense | 250 | 252 | 272 | 309 | 352 | 0.18 |
|  | Silent | 103,577 | 106,837 | 110,621 | 136,352 | 150,078 | 78.53 |
