# Supplementary material for: Analysis of Anasplatyrhynchos genome resequencing data reveals genetic signatures of artificial selection
Source: PLoS One. 2019 Feb 8;14(2):e0211908. doi: 10.1371/journal.pone.0211908 (PMC6368380; doi:10.1371/journal.pone.0211908)
Supplement: S6 Table — (DOCX) [file pone.0211908.s013.docx]

**S6 Table. Identified Indels for each duck population**

| Mutation types | | FTPD | LTPD | CMD | M | All | |
| --- | --- | --- | --- | --- | --- | --- | --- |
|  |  |  |  |  |  | Count | % |
| Classification by location | 5’ UTR | 48 | 50 | 51 | 66 | 69 | 0.03 |
|  | 3’ UTR | 1,161 | 1,178 | 1,200 | 1,422 | 1,548 | 0.71 |
|  | Exon | 2,024 | 2,034 | 2,074 | 2,170 | 2,324 | 1.06 |
|  | Intron | 161,186 | 165,282 | 168,873 | 199,576 | 215,606 | 98.20 |
| Classification by impact | High | 1,920 | 1,940 | 1,961 | 2,039 | 2,194 | 0.29 |
|  | Low | 1,130 | 1,184 | 1,184 | 1,376 | 1,487 | 0.20 |
|  | Moderate | 418 | 428 | 436 | 469 | 498 | 0.07 |
|  | Modifier | 553,746 | 567,632 | 580,166 | 679,108 | 740,210 | 99.44 |
