# Supplementary material for: Analysis of Anasplatyrhynchos genome resequencing data reveals genetic signatures of artificial selection
Source: PLoS One. 2019 Feb 8;14(2):e0211908. doi: 10.1371/journal.pone.0211908 (PMC6368380; doi:10.1371/journal.pone.0211908)
Supplement: S7 Table — (DOCX) [file pone.0211908.s014.docx]

**S7 Table. The genes under selection between artificial selection populations and their ancestor**

| Scaffold | Start | End | Ensembl ID | Gene name | Description |
| --- | --- | --- | --- | --- | --- |
| KB743335.1 | 600882 | 601670 | ENSAPLG00000000633 | LRRC18 | leucine rich repeat containing 18 |
| KB742931.1 | 3555478 | 3555807 | ENSAPLG00000001200 |  | Uncharacterized protein |
| KB743335.1 | 922094 | 983808 | ENSAPLG00000001314 | FRMPD2 | FERM and PDZ domain containing 2 |
| KB742785.1 | 718906 | 719028 | ENSAPLG00000001638 |  | Uncharacterized protein |
| KB743335.1 | 1117596 | 1122398 | ENSAPLG00000001902 | GDF10 | Bone morphogenetic protein 3b |
| KB742808.1 | 4568856 | 4656100 | ENSAPLG00000002432 | ZCCHC24 | Uncharacterized protein |
| KB743662.1 | 1241436 | 1359204 | ENSAPLG00000002607 | OCA2 | P protein |
| KB743739.1 | 521654 | 525734 | ENSAPLG00000002616 |  | Uncharacterized protein |
| KB743739.1 | 527927 | 531997 | ENSAPLG00000002828 | SMPDL3B | sphingomyelin phosphodiesterase, acid-like 3B |
| KB742479.1 | 1987407 | 1992041 | ENSAPLG00000002898 | C20orf85 | chromosome 20 open reading frame 85 |
| KB743739.1 | 535491 | 537292 | ENSAPLG00000002967 | XKR8 | XK, Kell blood group complex subunit-related family, member 8 |
| KB743739.1 | 541993 | 578161 | ENSAPLG00000002973 | EYA3 | EYA transcriptional coactivator and phosphatase 3 |
| KB743085.1 | 238355 | 244591 | ENSAPLG00000002977 | DBR1 | debranching RNA lariats 1 |
| KB743085.1 | 248911 | 308687 | ENSAPLG00000003230 | ARMC8 | armadillo repeat containing 8 |
| KB744306.1 | 807537 | 810767 | ENSAPLG00000003321 | FBXL22 | F-box/LRR-repeat protein 22 |
| KB744306.1 | 816863 | 841182 | ENSAPLG00000003323 | USP3 | ubiquitin specific peptidase 3 |
| KB742815.1 | 74323 | 376419 | ENSAPLG00000004522 | UTRN | utrophin |
| KB742390.1 | 138464 | 152641 | ENSAPLG00000005314 | LHFPL2 | lipoma HMGIC fusion partner-like 2 |
| KB742716.1 | 370308 | 373522 | ENSAPLG00000005797 | PPCS | phosphopantothenoylcysteine synthetase |
| KB742716.1 | 373371 | 385276 | ENSAPLG00000005847 | ZMYND12 | zinc finger, MYND-type containing 12 |
| KB742716.1 | 389995 | 401465 | ENSAPLG00000005941 | SLC2A1 | solute carrier family 2 (facilitated glucose transporter), member 1 |
| KB743543.1 | 339938 | 403234 | ENSAPLG00000006342 | LYST | Lysosomal-trafficking regulator |
| KB744955.1 | 37747 | 215580 | ENSAPLG00000007002 | HS6ST1 | heparan sulfate 6-O-sulfotransferase 1 |
| KB743948.1 | 10523 | 32116 | ENSAPLG00000007349 | ASUN | asunder spermatogenesis regulator |
| KB744638.1 | 1389 | 35742 | ENSAPLG00000007350 | FAM129A | family with sequence similarity 129, member A |
| KB742523.1 | 642235 | 690351 | ENSAPLG00000007352 | MEF2A | myocyte enhancer factor 2A |
| KB744638.1 | 39845 | 65973 | ENSAPLG00000007706 | EDEM3 | ER degradation enhancer, mannosidase alpha-like 3 |
| KB743948.1 | 37127 | 45371 | ENSAPLG00000007905 | FGFR1OP2 | FGFR1 oncogene partner 2 |
| KB743948.1 | 52669 | 67004 | ENSAPLG00000008163 | TM7SF3 | transmembrane 7 superfamily member 3 |
| KB742523.1 | 932722 | 1074839 | ENSAPLG00000008504 | IGF1R | insulin-like growth factor 1 receptor |
| KB743413.1 | 285075 | 462043 | ENSAPLG00000008508 | TRIO | trio Rho guanine nucleotide exchange factor |
| KB742432.1 | 186163 | 209871 | ENSAPLG00000009054 | STX7 | syntaxin 7 |
| KB742984.1 | 1613614 | 1664627 | ENSAPLG00000009070 | VAT1L | vesicle amine transport 1-like |
| KB742432.1 | 243937 | 286251 | ENSAPLG00000009110 | MOXD1 | monooxygenase, DBH-like 1 |
| KB742523.1 | 1083536 | 1093962 | ENSAPLG00000009905 | PGPEP1L | pyroglutamyl-peptidase I-like |
| KB742523.1 | 1111056 | 1132500 | ENSAPLG00000010042 | FAM169B | family with sequence similarity 169, member B |
| KB744518.1 | 5560 | 76235 | ENSAPLG00000010803 | RANBP10 | RAN binding protein 10 |
| KB742432.1 | 1257623 | 1293438 | ENSAPLG00000011115 | SAMD3 | sterile alpha motif domain containing 3 |
| KB742432.1 | 1296610 | 1372909 | ENSAPLG00000011121 | L3MBTL3 | l(3)mbt-like 3 (Drosophila) |
| KB744080.1 | 135397 | 226398 | ENSAPLG00000011715 | HCN1 | hyperpolarization activated cyclic nucleotide gated potassium channel 1 |
| KB743728.1 | 189204 | 197051 | ENSAPLG00000011816 | NOLC1 | nucleolar and coiled-body phosphoprotein 1 |
| KB743728.1 | 204454 | 210115 | ENSAPLG00000011857 |  | Uncharacterized protein |
| KB743004.1 | 446595 | 579671 | ENSAPLG00000011898 | CBLB | Cbl proto-oncogene B, E3 ubiquitin protein ligase |
| KB742527.1 | 805984 | 852598 | ENSAPLG00000011965 | MITF | microphthalmia-associated transcription factor |
| KB743728.1 | 220269 | 225974 | ENSAPLG00000011982 | ELOVL3 | ELOVL fatty acid elongase 3 |
| KB743728.1 | 231701 | 238209 | ENSAPLG00000012074 | PITX3 | paired-like homeodomain 3 |
| KB743922.1 | 58618 | 63286 | ENSAPLG00000012215 | ABI3 | ABI family, member 3 |
| KB743922.1 | 64989 | 65563 | ENSAPLG00000012604 | GNGT2 | Guanine nucleotide-binding protein subunit gamma |
| KB743922.1 | 77189 | 104884 | ENSAPLG00000012620 | IGF2BP1 | insulin-like growth factor 2 mRNA binding protein 1 |
| KB744663.1 | 139722 | 157684 | ENSAPLG00000012642 | PRG4 | proteoglycan 4 |
| KB744663.1 | 157898 | 201643 | ENSAPLG00000012730 | TPR | translocated promoter region, nuclear basket protein |
| KB742808.1 | 2447562 | 2755726 | ENSAPLG00000012993 | C10orf11 | chromosome 10 open reading frame 11 |
| KB742785.1 | 938090 | 1027864 | ENSAPLG00000013009 | PLA2G4A | phospholipase A2, group IVA (cytosolic, calcium-dependent) |
| KB742459.1 | 1585387 | 1614475 | ENSAPLG00000013015 | DUSP16 | dual specificity phosphatase 16 |
| KB743922.1 | 119027 | 123815 | ENSAPLG00000013204 |  | Uncharacterized protein |
| KB743922.1 | 125793 | 130919 | ENSAPLG00000013216 | SNF8 | Uncharacterized protein |
| KB743157.1 | 1210631 | 1274803 | ENSAPLG00000013219 | ZNF536 | zinc finger protein 536 |
| KB743922.1 | 132312 | 140894 | ENSAPLG00000013301 | UBE2Z | Ubiquitin-conjugating enzyme E2 Z |
| KB743922.1 | 143952 | 146171 | ENSAPLG00000013381 | ATP5G1 | ATP synthase, H+ transporting, mitochondrial Fo complex, subunit C1 (subunit 9) |
| KB743922.1 | 149526 | 157259 | ENSAPLG00000013396 | CALCOCO2 | calcium binding and coiled-coil domain 2 |
| KB742760.1 | 157884 | 275997 | ENSAPLG00000013475 | TULP4 | tubby like protein 4 |
| KB743922.1 | 189749 | 192075 | ENSAPLG00000013598 | HOXB13 | homeobox B13 |
| KB743922.1 | 233609 | 237855 | ENSAPLG00000013604 | HOXB9 | homeobox B9 |
| KB743922.1 | 246728 | 248314 | ENSAPLG00000013614 | HOXB8 | homeobox B8 |
| KB743922.1 | 250877 | 254643 | ENSAPLG00000013643 | HOXB7 | homeobox B7 |
| KB742785.1 | 1054076 | 1060584 | ENSAPLG00000013877 | PTGS2 | prostaglandin-endoperoxide synthase 2 (prostaglandin G/H synthase and cyclooxygenase) |
| KB744663.1 | 201872 | 216263 | ENSAPLG00000014252 | C1orf27 | chromosome 1 open reading frame 27 |
| KB743110.1 | 1735789 | 1751885 | ENSAPLG00000014701 | NAP1L4 | nucleosome assembly protein 1-like 4 |
| KB744663.1 | 221998 | 226757 | ENSAPLG00000014804 | PDC | phosducin |
| KB743944.1 | 2061657 | 2117379 | ENSAPLG00000014826 |  | Uncharacterized protein |
| KB743546.1 | 243724 | 342302 | ENSAPLG00000014895 | PREP | prolyl endopeptidase |
| KB743110.1 | 1758665 | 1787682 | ENSAPLG00000015062 | CARS | cysteinyl-tRNA synthetase |
| KB742833.1 | 1488645 | 1536985 | ENSAPLG00000015975 | FAM53A | family with sequence similarity 53, member A |
| KB743435.1 | 886740 | 936666 | ENSAPLG00000016252 | GYS2 | Glycogen |
| KB743335.1 | 326509 | 372165 | ENSAPLG00000016335 | ERCC6 | excision repair cross-complementation group 6 |
| KB743335.1 | 563928 | 619140 | ENSAPLG00000016371 |  | Uncharacterized protein |
| KB743435.1 | 951846 | 961751 | ENSAPLG00000016403 | LDHB | lactate dehydrogenase B |
| KB743335.1 | 811185 | 849617 | ENSAPLG00000016416 | ARHGAP22 | Rho GTPase activating protein 22 |
