# Supplementary material for: Analysis of Anasplatyrhynchos genome resequencing data reveals genetic signatures of artificial selection
Source: PLoS One. 2019 Feb 8;14(2):e0211908. doi: 10.1371/journal.pone.0211908 (PMC6368380; doi:10.1371/journal.pone.0211908)
Supplement: S8 Table — (DOCX) [file pone.0211908.s015.docx]

**S8 Table. The genes under selection between LTPD and FTPD**

| Scaffold | Start | End | Ensembl ID | Gene name | Description |
| --- | --- | --- | --- | --- | --- |
| KB742815.1 | 831616 | 832605 | ENSAPLG00000000729 | MAS1 | MAS proto-oncogene |
| KB742944.1 | 57459 | 57578 | ENSAPLG00000000812 |  | Uncharacterized protein |
| KB742944.1 | 96962 | 97936 | ENSAPLG00000000817 |  | Olfactory receptor |
| KB742944.1 | 123509 | 124582 | ENSAPLG00000000820 |  | Olfactory receptor |
| KB742944.1 | 131268 | 132227 | ENSAPLG00000000824 |  | Olfactory receptor |
| KB742944.1 | 142180 | 143091 | ENSAPLG00000000826 |  | Olfactory receptor |
| KB744353.1 | 13523 | 55363 | ENSAPLG00000001267 | IFT81 | intraflagellar transport 81 |
| KB742943.1 | 1069641 | 1070516 | ENSAPLG00000001617 | STX19 | Syntaxin-19 |
| KB742785.1 | 718906 | 719028 | ENSAPLG00000001638 |  | Uncharacterized protein |
| KB743510.1 | 63 | 12637 | ENSAPLG00000002168 | MAPK13 | mitogen-activated protein kinase 13 |
| KB744558.1 | 352125 | 471826 | ENSAPLG00000002327 | ZFHX3 | zinc finger homeobox 3 |
| KB743510.1 | 17400 | 34232 | ENSAPLG00000002603 | MAPK14 | mitogen-activated protein kinase 14 |
| KB742808.1 | 88896 | 118986 | ENSAPLG00000003394 | PDLIM1 | PDZ and LIM domain 1 |
| KB743289.1 | 888170 | 889123 | ENSAPLG00000003515 | TPPP2 | tubulin polymerization-promoting protein family member 2 |
| KB742808.1 | 132786 | 196120 | ENSAPLG00000003522 | SORBS1 | sorbin and SH3 domain containing 1 |
| KB743073.1 | 1287272 | 1380131 | ENSAPLG00000003528 | RABGAP1L | RAB GTPase activating protein 1-like |
| KB742473.1 | 156154 | 231972 | ENSAPLG00000003585 | ADGRL4 | adhesion G protein-coupled receptor L4 |
| KB743289.1 | 897975 | 938975 | ENSAPLG00000003590 | SYK | spleen tyrosine kinase |
| KB742840.1 | 1687610 | 1697626 | ENSAPLG00000003594 | CCDC62 | coiled-coil domain containing 62 |
| KB742479.1 | 2830560 | 2855261 | ENSAPLG00000003707 | RTFDC1 | replication termination factor 2 domain containing 1 |
| KB742840.1 | 1699111 | 1704759 | ENSAPLG00000003750 | DENR | density-regulated protein |
| KB742840.1 | 1705849 | 1710118 | ENSAPLG00000003776 | GPN3 | GPN-loop GTPase 3 |
| KB744033.1 | 881294 | 883954 | ENSAPLG00000003911 | PIK3IP1 | phosphoinositide-3-kinase interacting protein 1 |
| KB744033.1 | 899394 | 915645 | ENSAPLG00000003934 | PATZ1 | POZ (BTB) and AT hook containing zinc finger 1 |
| KB742840.1 | 1711479 | 1715358 | ENSAPLG00000004006 | ARPC3 | actin related protein 2/3 complex, subunit 3, 21kDa |
| KB742474.1 | 27060 | 264415 | ENSAPLG00000004055 | FAM172A | family with sequence similarity 172, member A |
| KB742479.1 | 2842471 | 2848761 | ENSAPLG00000004099 | GCNT7 | glucosaminyl (N-acetyl) transferase family member 7 |
| KB744033.1 | 925326 | 928805 | ENSAPLG00000004250 | DRG1 | Developmentally-regulated GTP-binding protein 1 |
| KB742840.1 | 1716341 | 1722935 | ENSAPLG00000004360 | ANAPC7 | Anaphase-promoting complex subunit 7 |
| KB742479.1 | 2857516 | 2867481 | ENSAPLG00000004370 |  | Uncharacterized protein |
| KB742479.1 | 2884493 | 2890609 | ENSAPLG00000004391 | CSTF1 | Cleavage stimulation factor 50 kDa subunit |
| KB744033.1 | 931756 | 951416 | ENSAPLG00000004503 | EIF4ENIF1 | eukaryotic translation initiation factor 4E nuclear import factor 1 |
| KB744353.1 | 129110 | 227471 | ENSAPLG00000004534 | KDM2B | lysine (K)-specific demethylase 2B |
| KB743145.1 | 3288045 | 3564230 | ENSAPLG00000004608 | SDK1 | sidekick cell adhesion molecule 1 |
| KB742479.1 | 2894251 | 2899525 | ENSAPLG00000004677 | AURKA | aurora kinase A |
| KB742479.1 | 2903024 | 2904181 | ENSAPLG00000004804 | FAM210B | family with sequence similarity 210, member B |
| KB742947.1 | 33364 | 234170 | ENSAPLG00000004846 |  | Uncharacterized protein |
| KB744353.1 | 246890 | 247658 | ENSAPLG00000004913 | ORAI1 | ORAI calcium release-activated calcium modulator 1 |
| KB742468.1 | 457711 | 565924 | ENSAPLG00000004925 | CACNA2D4 | calcium channel, voltage-dependent, alpha 2/delta subunit 4 |
| KB744353.1 | 251098 | 257038 | ENSAPLG00000004938 | MORN3 | MORN repeat containing 3 |
| KB744033.1 | 955679 | 980358 | ENSAPLG00000005212 | SFI1 | Sfi1 homolog, spindle assembly associated (yeast) |
| KB742446.1 | 771011 | 802429 | ENSAPLG00000005341 | RC3H1 | ring finger and CCCH-type domains 1 |
| KB743145.1 | 3577201 | 3590099 | ENSAPLG00000005773 |  | Uncharacterized protein |
| KB742446.1 | 827925 | 895180 | ENSAPLG00000005959 |  | Uncharacterized protein |
| KB742449.1 | 14121 | 22783 | ENSAPLG00000006230 | BPGM | 2,3-bisphosphoglycerate mutase |
| KB742449.1 | 37193 | 72863 | ENSAPLG00000006235 |  | Uncharacterized protein |
| KB744033.1 | 980543 | 991088 | ENSAPLG00000006251 |  | Uncharacterized protein |
| KB742947.1 | 193795 | 200134 | ENSAPLG00000006256 | ZNF276 | zinc finger protein 276 |
| KB742628.1 | 515575 | 545619 | ENSAPLG00000006384 | LCORL | ligand dependent nuclear receptor corepressor-like |
| KB742947.1 | 200224 | 233601 | ENSAPLG00000006484 | FANCA | Fanconi anemia, complementation group A |
| KB742628.1 | 555348 | 557201 | ENSAPLG00000006658 |  | Uncharacterized protein |
| KB742628.1 | 574477 | 598681 | ENSAPLG00000006687 | NCAPG | non-SMC condensin I complex, subunit G |
| KB742793.1 | 21941 | 108300 | ENSAPLG00000006759 | APBB2 | amyloid beta (A4) precursor protein-binding, family B, member 2 |
| KB742588.1 | 923146 | 946309 | ENSAPLG00000006989 | RHOT1 | ras homolog family member T1 |
| KB742628.1 | 607242 | 645525 | ENSAPLG00000007157 | FAM184B | family with sequence similarity 184, member B |
| KB742815.1 | 850473 | 896528 | ENSAPLG00000007173 | IGF2R | Cation-independent mannose-6-phosphate receptor |
| KB742588.1 | 990758 | 1012813 | ENSAPLG00000007263 | RHBDL3 | rhomboid, veinlet-like 3 (Drosophila) |
| KB743896.1 | 1051311 | 1058651 | ENSAPLG00000007430 | INSIG1 | insulin induced gene 1 |
| KB742815.1 | 899712 | 911167 | ENSAPLG00000007770 | SLC22A1 | Uncharacterized protein |
| KB742386.1 | 227991 | 306553 | ENSAPLG00000008044 | UST | Uncharacterized protein |
| KB743177.1 | 207180 | 239449 | ENSAPLG00000008174 |  | Uncharacterized protein |
| KB744033.1 | 254188 | 326654 | ENSAPLG00000009657 | CIT | citron rho-interacting serine/threonine kinase |
| KB743686.1 | 1436913 | 1593403 | ENSAPLG00000010566 | RANBP17 | RAN binding protein 17 |
| KB742887.1 | 2451484 | 2512660 | ENSAPLG00000010662 | LMO3 | LIM domain only protein 3 |
| KB744033.1 | 353548 | 396735 | ENSAPLG00000010709 | CCDC64 | coiled-coil domain containing 64 |
| KB742887.1 | 2578424 | 2585344 | ENSAPLG00000010726 | MGST1 | Microsomal glutathione S-transferase 1 |
| KB742944.1 | 27891 | 41290 | ENSAPLG00000010729 | ACADM | acyl-CoA dehydrogenase, C-4 to C-12 straight chain |
| KB742887.1 | 2622830 | 2650597 | ENSAPLG00000010794 | SLC15A5 | Peptide/histidine transporter |
| KB742887.1 | 2704894 | 2747279 | ENSAPLG00000010934 | DERA | deoxyribose-phosphate aldolase (putative) |
| KB742887.1 | 2758187 | 2765563 | ENSAPLG00000011004 | STRAP | serine/threonine kinase receptor associated protein |
| KB742542.1 | 438730 | 452971 | ENSAPLG00000011060 | WDR75 | WD repeat domain 75 |
| KB742944.1 | 155349 | 158655 | ENSAPLG00000011277 |  | Uncharacterized protein |
| KB742639.1 | 1015152 | 1152076 | ENSAPLG00000011519 | MRAP2 | Uncharacterized protein |
| KB742639.1 | 1095207 | 1134296 | ENSAPLG00000011565 | CEP162 | centrosomal protein 162kDa |
| KB742943.1 | 1041026 | 1085175 | ENSAPLG00000011691 | ARL13B | ADP-ribosylation factor-like 13B |
| KB742855.1 | 166405 | 187886 | ENSAPLG00000011907 | IL13RA2 | interleukin 13 receptor, alpha 2 |
| KB743583.1 | 153560 | 155822 | ENSAPLG00000011937 | NPVF | neuropeptide VF precursor |
| KB743583.1 | 172219 | 192497 | ENSAPLG00000012025 | C7orf31 | chromosome 7 open reading frame 31 |
| KB742855.1 | 202212 | 243045 | ENSAPLG00000012164 | HTR2C | 5-hydroxytryptamine receptor 2C |
| KB743583.1 | 197302 | 198862 | ENSAPLG00000012192 | CYC | Cytochrome c |
| KB742734.1 | 1443431 | 1445686 | ENSAPLG00000012252 | BATF3 | basic leucine zipper transcription factor, ATF-like 3 |
| KB742734.1 | 1474247 | 1476861 | ENSAPLG00000012304 | ATF3 | Cyclic AMP-dependent transcription factor ATF-3 |
| KB743158.1 | 751184 | 759253 | ENSAPLG00000012840 |  | Uncharacterized protein |
| KB743358.1 | 72372 | 87905 | ENSAPLG00000012849 | WFS1 | Wolfram syndrome 1 (wolframin) |
| KB743956.1 | 374342 | 497942 | ENSAPLG00000012910 | ZFHX4 | zinc finger homeobox 4 |
| KB744681.1 | 28255 | 104224 | ENSAPLG00000013256 | CAMSAP2 | calmodulin regulated spectrin-associated protein family, member 2 |
| KB742888.1 | 622536 | 759832 | ENSAPLG00000013665 | AGBL1 | ATP/GTP binding protein-like 1 |
| KB742932.1 | 441355 | 484880 | ENSAPLG00000013878 | NFKB1 | nuclear factor of kappa light polypeptide gene enhancer in B-cells 1 |
| KB742395.1 | 545012 | 586221 | ENSAPLG00000014002 | NCAPG2 | non-SMC condensin II complex, subunit G2 |
| KB742840.1 | 1225355 | 1278283 | ENSAPLG00000014113 | DNAH10 | dynein, axonemal, heavy chain 10 |
| KB742840.1 | 1281714 | 1298105 | ENSAPLG00000014518 | ATP6V0A2 | ATPase, H+ transporting, lysosomal V0 subunit a2 |
| KB742840.1 | 1298359 | 1307261 | ENSAPLG00000014650 | TCTN2 | tectonic family member 2 |
| KB742932.1 | 491751 | 529529 | ENSAPLG00000014676 | MANBA | mannosidase, beta A, lysosomal |
| KB742840.1 | 1310017 | 1314163 | ENSAPLG00000014734 | GTF2H3 | general transcription factor IIH, polypeptide 3, 34kDa |
| KB742840.1 | 1314575 | 1318151 | ENSAPLG00000014771 | EIF2B1 | eukaryotic translation initiation factor 2B, subunit 1 alpha, 26kDa |
| KB742840.1 | 1318327 | 1324532 | ENSAPLG00000015032 | DDX55 | DEAD (Asp-Glu-Ala-Asp) box polypeptide 55 |
| KB744722.1 | 917820 | 921231 | ENSAPLG00000015630 | LRRC8B | Leucine-rich repeat-containing protein 8B |
| KB744722.1 | 952058 | 957223 | ENSAPLG00000015632 | LRRC8C | leucine rich repeat containing 8 family, member C |
| KB743342.1 |  |  | ENSAPLG00000016236 | PRKACB | Uncharacterized protein |
