# Supplementary material for: Analysis of Anasplatyrhynchos genome resequencing data reveals genetic signatures of artificial selection
Source: PLoS One. 2019 Feb 8;14(2):e0211908. doi: 10.1371/journal.pone.0211908 (PMC6368380; doi:10.1371/journal.pone.0211908)
Supplement: S9 Table — (DOCX) [file pone.0211908.s016.docx]

**S9 Table. The functional enrichment analysis for genes under selection between M and AS**

| Term | Description | Gene counts | Pvalue | Gene ID | Gene names |
| --- | --- | --- | --- | --- | --- |
| GO:0030318 | Melanocyte differentiation | 3 (60) | 0.0002 | ENSAPLG00000002607  ENSAPLG00000011965  ENSAPLG00000012993 | OCA2  MITF  C10orf11 |
| GO:0032509 | Endosome transport via multivesicular body sorting pathway | 2 (60) | 0.0002 | ENSAPLG00000006342  ENSAPLG00000013216 | LYST  SNF8 |
| GO:0043473 | Pigmentation | 4 (60) | 0.0004 | ENSAPLG00000002607  ENSAPLG00000006342  ENSAPLG00000011965  ENSAPLG00000012993 | OCA2  LYST MITF  C10orf11 |
| GO:0050931 | Pigment cell differentiation | 3 (60) | 0.0005 | ENSAPLG00000002607  ENSAPLG00000011965  ENSAPLG00000012993 | OCA2  MITF  C10orf11 |
| GO:0048066 | Developmental pigmentation | 3 (60) | 0.0009 | ENSAPLG00000002607  ENSAPLG00000011965  ENSAPLG00000012993 | OCA2  MITF  C10orf11 |
| GO:0006997 | Nucleus organization | 3 (60) | 0.0041 | ENSAPLG00000007349  ENSAPLG00000011816  ENSAPLG00000012730 | ASUN  NOLC1  TPR |
| GO:0044249 | Cellular biosynthetic process | 26 (60) | 0.0042 | ENSAPLG00000001200  ENSAPLG00000002607  ENSAPLG00000002616  ENSAPLG00000007002  ENSAPLG00000007350  ENSAPLG00000007352  ENSAPLG00000008504  ENSAPLG00000011121  ENSAPLG00000011965  ENSAPLG00000011982  ENSAPLG00000012074  ENSAPLG00000012620  ENSAPLG00000012642  ENSAPLG00000012730  ENSAPLG00000013009  ENSAPLG00000013216  ENSAPLG00000013219  ENSAPLG00000013381  ENSAPLG00000013598  ENSAPLG00000013604  ENSAPLG00000013614  ENSAPLG00000013643  ENSAPLG00000013877  ENSAPLG00000015062  ENSAPLG00000016252  ENSAPLG00000016335 | OCA2  HS6ST1  FAM129A  MEF2A  IGF1R  L3MBTL3  MITF  ELOVL3  PITX3  IGF2BP1  PRG4  TPR  PLA2G4A  SNF8  ZNF536  ATP5G1  HOXB13  HOXB9  HOXB8  HOXB7  PTGS2  CARS  GYS2  ERCC6 |
| GO:1901570 | Fatty acid derivative biosynthetic process | 2 (60) | 0.0049 | ENSAPLG00000013009 ENSAPLG00000013877 | PLA2G4A  PTGS2 |
| GO:0046456 | Icosanoid biosynthetic process | 2 (60) | 0.0049 | ENSAPLG00000013009  ENSAPLG00000013877 | PLA2G4A  PTGS2 |
| GO:1901576 | Organic substance biosynthetic process | 26 (60) | 0.0056 | ENSAPLG00000001200  ENSAPLG00000002607  ENSAPLG00000002616  ENSAPLG00000007002  ENSAPLG00000007350  ENSAPLG00000007352  ENSAPLG00000008504  ENSAPLG00000011121  ENSAPLG00000011965  ENSAPLG00000011982  ENSAPLG00000012074  ENSAPLG00000012620  ENSAPLG00000012642  ENSAPLG00000012730  ENSAPLG00000013009  ENSAPLG00000013216  ENSAPLG00000013219  ENSAPLG00000013381  ENSAPLG00000013598  ENSAPLG00000013604  ENSAPLG00000013614  ENSAPLG00000013643  ENSAPLG00000013877  ENSAPLG00000015062  ENSAPLG00000016252  ENSAPLG00000016335 | OCA2  HS6ST1  FAM129A  MEF2A  IGF1R  L3MBTL3  MITF  ELOVL3  PITX3  IGF2BP1  PRG4  TPR  PLA2G4A  SNF8  ZNF536  ATP5G1  HOXB13  HOXB9  HOXB8  HOXB7  PTGS2  CARS  GYS2  ERCC6 |
| GO:0000189 | MAPK import into nucleus | 1 (60) | 0.0056 | ENSAPLG00000012730 | TPR |
| GO:0010797 | Regulation of multivesicular body size involved in endosome transport | 1 (60) | 0.0056 | ENSAPLG00000013216 | SNF8 |
| GO:0070782 | Phosphatidylserine exposure on apoptotic cell surface | 1 (60) | 0.0056 | ENSAPLG00000002967 | XKR8 |
| GO:0010847 | Regulation of chromatin assembly | 1 (60) | 0.0056 | ENSAPLG00000012730 | TPR |
| GO:0010610 | Regulation of mrna stability involved in response to stress | 1 (60) | 0.0056 | ENSAPLG00000012620 | IGF2BP1 |
| GO:0031453 | Positive regulation of heterochromatin assembly | 1 (60) | 0.0056 | ENSAPLG00000012730 | TPR |
| GO:0031445 | Regulation of heterochromatin assembly | 1 (60) | 0.0056 | ENSAPLG00000012730 | TPR |
| GO:0006404 | RNA import into nucleus | 1 (60) | 0.0056 | ENSAPLG00000012730 | TPR |
| GO:0031990 | Mrna export from nucleus in response to heat stress | 1 (60) | 0.0056 | ENSAPLG00000012730 | TPR |
| GO:0032240 | Negative regulation of nucleobase-containing compound transport | 1 (60) | 0.0056 | ENSAPLG00000012730 | TPR |
| GO:0061635 | Regulation of protein complex stability | 1 (60) | 0.0056 | ENSAPLG00000013216 | SNF8 |
| GO:1902683 | Regulation of receptor localization to synapse | 1 (60) | 0.0056 | ENSAPLG00000009054 | STX7 |
| GO:1902685 | Positive regulation of receptor localization to synapse | 1 (60) | 0.0056 | ENSAPLG00000009054 | STX7 |
| GO:0022013 | Pallium cell proliferation in forebrain | 1 (60) | 0.0056 | ENSAPLG00000012620 | IGF2BP1 |
| GO:0051389 | Inactivation of MAPKK activity | 1 (60) | 0.0056 | ENSAPLG00000008504 | IGF1R |
| GO:0015700 | Arsenite transport | 1 (60) | 0.0056 | ENSAPLG00000002607 |  |
| GO:0046832 | Negative regulation of RNA export from nucleus | 1 (60) | 0.0056 | ENSAPLG00000012730 | TPR |
| GO:0070375 | ERK5 cascade | 1 (60) | 0.0056 | ENSAPLG00000007352 | MEF2A |
| GO:0060563 | Neuroepithelial cell differentiation | 3 (60) | 0.0067 | ENSAPLG00000002607  ENSAPLG00000011965  ENSAPLG00000012993 | OCA2  MITF  C10orf11 |
| GO:0009058 | Biosynthetic process | 26 (60) | 0.0075 | ENSAPLG00000001200  ENSAPLG00000002607  ENSAPLG00000002616  ENSAPLG00000007002  ENSAPLG00000007350  ENSAPLG00000007352  ENSAPLG00000008504  ENSAPLG00000011121  ENSAPLG00000011965  ENSAPLG00000011982  ENSAPLG00000012074  ENSAPLG00000012620  ENSAPLG00000012642  ENSAPLG00000012730  ENSAPLG00000013009  ENSAPLG00000013216  ENSAPLG00000013219  ENSAPLG00000013381  ENSAPLG00000013598  ENSAPLG00000013604  ENSAPLG00000013614  ENSAPLG00000013643  ENSAPLG00000013877  ENSAPLG00000015062  ENSAPLG00000016252  ENSAPLG00000016335 | OCA2  HS6ST1  FAM129A  MEF2A  IGF1R  L3MBTL3  MITF  ELOVL3  PITX3  IGF2BP1  PRG4  TPR  PLA2G4A  SNF8  ZNF536  ATP5G1  HOXB13  HOXB9  HOXB8  HOXB7  PTGS2  CARS  GYS2  ERCC6 |
| GO:0045022 | Early endosome to late endosome transport | 2 (60) | 0.0078 | ENSAPLG00000006342  ENSAPLG00000013216 | LYST  SNF8 |
| GO:0051054 | Positive regulation of DNA metabolic process | 3 (60) | 0.0087 | ENSAPLG00000002973  ENSAPLG00000008504  ENSAPLG00000012730 | EYA3  IGF1R  TPR |
| GO:0060740 | Prostate gland epithelium morphogenesis | 2 (60) | 0.0098 | ENSAPLG00000008504  ENSAPLG00000013598 | IGF1R  HOXB13 |
