# Supplementary material for: Analysis of Anasplatyrhynchos genome resequencing data reveals genetic signatures of artificial selection
Source: PLoS One. 2019 Feb 8;14(2):e0211908. doi: 10.1371/journal.pone.0211908 (PMC6368380; doi:10.1371/journal.pone.0211908)
Supplement: S10 Table — (DOCX) [file pone.0211908.s017.docx]

**S10 Table. The functional enrichment analysis for genes under selection between FTPD and LTPD**

| Term | Description | Gene counts | Pvalue | Gene ID | Gene names |
| --- | --- | --- | --- | --- | --- |
| GO:0050906 | Detection of stimulus involved in sensory perception | 5 (76) | 0.0013 | ENSAPLG00000000817  ENSAPLG00000000820  ENSAPLG00000000824  ENSAPLG00000000826  ENSAPLG00000004925 | Olfactory receptor （OF）  OF  OF  OF  CACNA2D4 |
| GO:0045598 | Regulation of fat cell differentiation | 4 (76) | 0.0015 | ENSAPLG00000002603  ENSAPLG00000007430  ENSAPLG00000010662  ENSAPLG00000012164 | MAPK14  INSIG1  LMO3  HTR2C |
| GO:0050911 | Detection of chemical stimulus involved in sensory perception of smell | 4 (76) | 0.0017 | ENSAPLG00000000817  ENSAPLG00000000820  ENSAPLG00000000824  ENSAPLG00000000826 | OF  OF  OF  OF |
| GO:0045600 | Positive regulation of fat cell differentiation | 3 (76) | 0.002 | ENSAPLG00000002603  ENSAPLG00000010662  ENSAPLG00000012164 | MAPK14  LMO3  HTR2C |
| GO:0045444 | Fat cell differentiation | 5 (76) | 0.0021 | ENSAPLG00000002603  ENSAPLG00000007430  ENSAPLG00000010662  ENSAPLG00000012164  ENSAPLG00000015632 | MAPK14  INSIG1  LMO3  HTR2C  LRRC8C |
| GO:0050907 | Detection of chemical stimulus involved in sensory perception | 4 (76) | 0.0025 | ENSAPLG00000000817  ENSAPLG00000000820  ENSAPLG00000000824  ENSAPLG00000000826 | OF  OF  OF  OF |
| GO:0045663 | Positive regulation of myoblast differentiation | 3 (76) | 0.0026 | ENSAPLG00000002327  ENSAPLG00000002603  ENSAPLG00000007173 | ZFHX3  MAPK14  IGF2R |
| GO:1901224 | Positive regulation of NIK/NF-kappab signaling | 2 (76) | 0.0031 | ENSAPLG00000000729  ENSAPLG00000005341 | MAS1  RC3H1 |
| GO:0007608 | Sensory perception of smell | 4 (76) | 0.0032 | ENSAPLG00000000817  ENSAPLG00000000820  ENSAPLG00000000824  ENSAPLG00000000826 | OF  OF  OF  OF |
| GO:0035994 | Response to muscle stretch | 2 (76) | 0.0037 | ENSAPLG00000002603  ENSAPLG00000013878 | MAPK14  NFKB1 |
| GO:0043304 | Regulation of mast cell degranulation | 2(76) | 0.0037 | ENSAPLG00000003590  ENSAPLG00000011907 | SYK  IL13RA2 |
| GO:0033006 | Regulation of mast cell activation involved in immune response | 2 (76) | 0.0042 | ENSAPLG00000003590  ENSAPLG00000011907 | SYK  IL13RA2 |
| GO:0009593 | Detection of chemical stimulus | 4 (76) | 0.0045 | ENSAPLG00000000817  ENSAPLG00000000820  ENSAPLG00000000824  ENSAPLG00000000826 | OF  OF  OF  OF |
| GO:0005976 | Polysaccharide metabolic process | 3 (76) | 0.0049 | ENSAPLG00000010729  ENSAPLG00000013878  ENSAPLG00000014676 | MAS1  NFKB1  MANBA |
| GO:0031281 | Positive regulation of cyclase activity | 2 (76) | 0.0055 | ENSAPLG00000002603  ENSAPLG00000012849 | MAPK14  WFS1 |
| GO:1901222 | Regulation of NIK/NF-kappab signaling | 2 (76) | 0.0055 | ENSAPLG00000000729  ENSAPLG00000005341 | MAS1  RC3H1 |
| GO:0051606 | Detection of stimulus | 5 (76) | 0.0056 | ENSAPLG00000000817  ENSAPLG00000000820  ENSAPLG00000000824  ENSAPLG00000000826  ENSAPLG00000004925 | OF  OF  OF  OF  CACNA2D4 |
| GO:0010894 | Negative regulation of steroid biosynthetic process | 2 (76) | 0.0063 | ENSAPLG00000007430  ENSAPLG00000013878 | INSIG1  NFKB1 |
| GO:0033003 | Regulation of mast cell activation | 2 (76) | 0.0063 | ENSAPLG00000003590  ENSAPLG00000011907 | SYK  IL13RA2 |
| GO:0045939 | Negative regulation of steroid metabolic process | 2 (76) | 0.0063 | ENSAPLG00000007430  ENSAPLG00000013878 | INSIG1  NFKB1 |
| GO:0031326 | Regulation of cellular biosynthetic process | 23 (76) | 0.0069 | ENSAPLG00000002327  ENSAPLG00000002603  ENSAPLG00000003394  ENSAPLG00000003590  ENSAPLG00000003594  ENSAPLG00000003934  ENSAPLG00000004503  ENSAPLG00000004534  ENSAPLG00000005212  ENSAPLG00000006384  ENSAPLG00000006759  ENSAPLG00000007430  ENSAPLG00000010709  ENSAPLG00000010729  ENSAPLG00000011004  ENSAPLG00000011519  ENSAPLG00000012164  ENSAPLG00000012252  ENSAPLG00000012304  ENSAPLG00000012849  ENSAPLG00000012910  ENSAPLG00000013878  ENSAPLG00000014734 | ZFHX3  MAPK14  PDLIM1  SYK  CCDC62  PATZ1  EIF4ENIF1  KDM2B  SFI1  LCORL  APBB2  INSIG1  CCDC64  STRAP  MRAP2  HTR2C  BATF3  ATF3  WFS1  ZFHX4  NFKB1  GTF2H3 |
| GO:0002886 | Regulation of myeloid leukocyte mediated immunity | 2 (76) | 0.007 | ENSAPLG00000003590  ENSAPLG00000011907 | SYK  IL13RA2 |
| GO:0042253 | Granulocyte macrophage colony-stimulating factor biosynthetic process | 1 (76) | 0.0071 | ENSAPLG00000003590 | SYK |
| GO:0042223 | Interleukin-3 biosynthetic process | 1 (76) | 0.0071 | ENSAPLG00000003590 | SYK |
| GO:0071226 | Cellular response to molecule of fungal origin | 1 (76) | 0.0071 | ENSAPLG00000003590 | SYK |
| GO:0070843 | Misfolded protein transport | 1 (76) | 0.0071 | ENSAPLG00000012849 | WFS1 |
| GO:0070844 | Polyubiquitinated protein transport | 1 (76) | 0.0071 | ENSAPLG00000012849 | WFS1 |
| GO:0070845 | Polyubiquitinated misfolded protein transport | 1 (76) | 0.0071 | ENSAPLG00000012849 | WFS1 |
| GO:1900195 | Positive regulation of oocyte maturation | 1 (76) | 0.0071 | ENSAPLG00000004677 | AURKA |
| GO:0006982 | Response to lipid hydroperoxide | 1 (76) | 0.0071 | ENSAPLG00000010726 | MGST1 |
| GO:0002238 | Response to molecule of fungal origin | 1 (76) | 0.0071 | ENSAPLG00000003590 | SYK |
| GO:2000324 | Positive regulation of glucocorticoid receptor signaling pathway | 1(76) | 0.0071 | ENSAPLG00000010662 | LMO3 |
| GO:0032752 | Positive regulation of interleukin-3 production | 1 (76) | 0.0071 | ENSAPLG00000003590 | SYK |
| GO:0007208 | Phospholipase C-activating serotonin receptor signaling pathway | 1 (76) | 0.0071 | ENSAPLG00000012164 | HTR2C |
| GO:0045083 | Negative regulation of interleukin-12 biosynthetic process | 1 (76) | 0.0071 | ENSAPLG00000013878 | NFKB1 |
| GO:0032672 | Regulation of interleukin-3 production | 1 (76) | 0.0071 | ENSAPLG00000003590 | SYK |
| GO:0045399 | Regulation of interleukin-3 biosynthetic process | 1 (76) | 0.0071 | ENSAPLG00000003590 | SYK |
| GO:0045423 | Regulation of granulocyte macrophage colony-stimulating factor biosynthetic process | 1 (76) | 0.0071 | ENSAPLG00000003590 | SYK |
| GO:0045425 | Positive regulation of granulocyte macrophage colony-stimulating factor biosynthetic process | 1 (76) | 0.0071 | ENSAPLG00000003590 | SYK |
| GO:0045401 | Positive regulation of interleukin-3 biosynthetic process | 1 (76) | 0.0071 | ENSAPLG00000003590 | SYK |
| GO:0021993 | Initiation of neural tube closure | 1 (76) | 0.0071 | ENSAPLG00000004534 | KDM2B |
| GO:0055107 | Golgi to secretory granule transport | 1 (76) | 0.0071 | ENSAPLG00000010709 | CCDC64 |
| GO:0046355 | Mannan catabolic process | 1 (76) | 0.0071 | ENSAPLG00000014676 | MANBA |
| GO:0010412 | Mannan metabolic process | 1 (76) | 0.0071 | ENSAPLG00000014676 | MANBA |
| GO:0071316 | Cellular response to nicotine | 1 (76) | 0.0071 | ENSAPLG00000013878 | NFKB1 |
| GO:0071449 | Cellular response to lipid hydroperoxide | 1 (76) | 0.0071 | ENSAPLG00000010726 | MGST1 |
| GO:0007606 | Sensory perception of chemical stimulus | 4 (76) | 0.0073 | ENSAPLG00000000817  ENSAPLG00000000820  ENSAPLG00000000824  ENSAPLG00000000826 | OF  OF  OF  OF |
| GO:0043300 | Regulation of leukocyte degranulation | 2 (76) | 0.0078 | ENSAPLG00000003590  ENSAPLG00000011907 | SYK  IL13RA2 |
| GO:0043303 | Mast cell degranulation | 2 (76) | 0.0078 | ENSAPLG00000003590  ENSAPLG00000011907 | SYK  IL13RA2 |
| GO:0002532 | Production of molecular mediator involved in inflammatory response | 2 (76) | 0.0078 | ENSAPLG00000002603  ENSAPLG00000003590 | MAPK14  SYK |
| GO:0038061 | NIK/NF-kappab signaling | 2 (76) | 0.0078 | ENSAPLG00000000729  ENSAPLG00000005341 | MAS1  RC3H1 |
| GO:0009889 | Regulation of biosynthetic process | 23 (76) | 0.008 | ENSAPLG00000002327  ENSAPLG00000002603  ENSAPLG00000003394  ENSAPLG00000003590  ENSAPLG00000003594  ENSAPLG00000003934  ENSAPLG00000004503  ENSAPLG00000004534  ENSAPLG00000005212  ENSAPLG00000006384  ENSAPLG00000006759  ENSAPLG00000007430  ENSAPLG00000010709  ENSAPLG00000010729  ENSAPLG00000011004  ENSAPLG00000011519  ENSAPLG00000012164  ENSAPLG00000012252  ENSAPLG00000012304  ENSAPLG00000012849  ENSAPLG00000012910  ENSAPLG00000013878  ENSAPLG00000014734 | ZFHX3  MAPK14  PDLIM1  SYK  CCDC62  PATZ1  EIF4ENIF1  KDM2B  SFI1  LCORL  APBB2  INSIG1  CCDC64  STRAP  MRAP2  HTR2C  BATF3  ATF3  WFS1  ZFHX4  NFKB1  GTF2H3 |
| GO:0002448 | Mast cell mediated immunity | 2 (76) | 0.0086 | ENSAPLG00000003590  ENSAPLG00000011907 | SYK  IL13RA2 |
| GO:0002279 | Mast cell activation involved in immune response | 2 (76) | 0.0086 | ENSAPLG00000003590  ENSAPLG00000011907 | SYK  IL13RA2 |
| GO:1903305 | Regulation of regulated secretory pathway | 2 (76) | 0.0086 | ENSAPLG00000003590  ENSAPLG00000011907 | SYK  IL13RA2 |
| GO:0070372 | Regulation of ERK1 and ERK2 cascade | 4 (76) | 0.0091 | ENSAPLG00000003590  ENSAPLG00000010662  ENSAPLG00000012164  ENSAPLG00000012304 | SYK  LMO3  HTR2C  ATF3 |
