# Supplementary material for: Analysis of Anasplatyrhynchos genome resequencing data reveals genetic signatures of artificial selection
Source: PLoS One. 2019 Feb 8;14(2):e0211908. doi: 10.1371/journal.pone.0211908 (PMC6368380; doi:10.1371/journal.pone.0211908)
Supplement: S11 Table — (DOCX) [file pone.0211908.s018.docx]

**S11 Table. The top 20 selected genes in the M-AS comparison**

| Gene name | scaffold | start | end | snp12 | Fst | Snp1 | Hp-M | Snp2 | Hp-AS |
| --- | --- | --- | --- | --- | --- | --- | --- | --- | --- |
| HCN1 | KB744080.1 | 140000 | 180000 | 84 | 0.78 | 47 | 0.35 | 37 | 0.16 |
| HOXB9 | KB743922.1 | 200000 | 240000 | 59 | 0.66 | 48 | 0.36 | 29 | 0.10 |
| HOXB7 | KB743922.1 | 220000 | 260000 | 50 | 0.64 | 36 | 0.42 | 26 | 0.11 |
| HOXB8 | KB743922.1 | 220000 | 260000 | 50 | 0.64 | 36 | 0.42 | 26 | 0.11 |
| PTGS2 | KB742785.1 | 1020000 | 1060000 | 44 | 0.63 | 40 | 0.23 | 8 | 0.09 |
| MITF | KB742527.1 | 800000 | 840000 | 235 | 0.62 | 201 | 0.33 | 61 | 0.30 |
| C10orf11 | KB742808.1 | 2420000 | 2460000 | 60 | 0.62 | 56 | 0.35 | 53 | 0.17 |
| PDC | KB744663.1 | 200000 | 240000 | 90 | 0.59 | 90 | 0.27 | 20 | 0.08 |
| C1orf27 | KB744663.1 | 180000 | 220000 | 73 | 0.59 | 78 | 0.29 | 13 | 0.07 |
| ARMC8 | KB743085.1 | 240000 | 280000 | 223 | 0.58 | 264 | 0.36 | 155 | 0.07 |
| DBR1 | KB743085.1 | 240000 | 280000 | 223 | 0.58 | 264 | 0.36 | 155 | 0.07 |
| LHFPL2 | KB742390.1 | 100000 | 140000 | 162 | 0.58 | 107 | 0.41 | 115 | 0.17 |
| NOLC1 | KB743728.1 | 180000 | 220000 | 432 | 0.57 | 403 | 0.39 | 343 | 0.15 |
|  | KB743728.1 | 180000 | 220000 | 432 | 0.57 | 403 | 0.39 | 343 | 0.15 |
| ATP5G1 | KB743922.1 | 120000 | 160000 | 68 | 0.56 | 78 | 0.36 | 60 | 0.10 |
| CALCOCO2 | KB743922.1 | 120000 | 160000 | 68 | 0.56 | 78 | 0.36 | 60 | 0.10 |
| ELOVL3 | KB743728.1 | 200000 | 240000 | 468 | 0.56 | 394 | 0.39 | 346 | 0.14 |
| PITX3 | KB743728.1 | 200000 | 240000 | 468 | 0.56 | 394 | 0.39 | 346 | 0.14 |
|  | KB742785.1 | 680000 | 720000 | 69 | 0.55 | 68 | 0.32 | 13 | 0.14 |
| PLA2G4A | KB742785.1 | 900000 | 940000 | 80 | 0.54 | 79 | 0.41 | 11 | 0.08 |
