# Supplementary material for: Analysis of Anasplatyrhynchos genome resequencing data reveals genetic signatures of artificial selection
Source: PLoS One. 2019 Feb 8;14(2):e0211908. doi: 10.1371/journal.pone.0211908 (PMC6368380; doi:10.1371/journal.pone.0211908)
Supplement: S12 Table — (DOCX) [file pone.0211908.s019.docx]

**S12 Table. The annotation of Indels identified in the M-AS comparison**

| Gene name | Scaffolds | Position | Reference | Alternative | Information | Effect level | Mutation description |
| --- | --- | --- | --- | --- | --- | --- | --- |
| *PREP* | KB743546.1 | 326363 | AT | A | splice_donor_variant&intron_variant | HIGH | c.666+1delA |
| *HCN1* | KB744080.1 | 136444 | GGC | G | splice_acceptor_variant&splice_region_variant&intron_variant | HIGH | c.1357-2_1357-1delGC |
| *FAM53A* | KB742833.1 | 1526751 | C | CG | frameshift_variant | HIGH | c.400_401insC |
| *FAM53A* | KB742833.1 | 1526753 | G | GC | frameshift_variant | HIGH | c.398dupG |
| *IGF1R* | KB742523.1 | 932918 | C | CT | frameshift_variant | HIGH | c.74dupT |
| *CBLB* | KB743004.1 | 446669 | C | CA | frameshift_variant | HIGH | c.76dupA |
| *CBLB* | KB743004.1 | 446673 | A | AG | frameshift_variant | HIGH | c.80dupG |
| *CBLB* | KB743004.1 | 446680 | GCC | G | frameshift_variant | HIGH | c.89_90delCC |
| *ENSAPLG00000001314* | KB743335.1 | 930391 | CA | C | frameshift_variant | HIGH | c.1011delA |
| *HS6ST1* | KB744955.1 | 37980 | CT | C | frameshift_variant | HIGH | c.236delT |
