# Supplementary material for: Analysis of Anasplatyrhynchos genome resequencing data reveals genetic signatures of artificial selection
Source: PLoS One. 2019 Feb 8;14(2):e0211908. doi: 10.1371/journal.pone.0211908 (PMC6368380; doi:10.1371/journal.pone.0211908)
Supplement: S13 Table — (DOCX) [file pone.0211908.s020.docx]

**S13 Table. The top 20 selected genes in the FTPD-LTPD comparison**

| Gene name | Scaffold | Start | End | snp12 | Fst | Snp  -ZF | Hp  -ZF | Snp  -ZM | Hp  -ZM |
| --- | --- | --- | --- | --- | --- | --- | --- | --- | --- |
|  | KB745151.1 | 0 | 40000 | 11 | 0.61 | 9 | 0.23 | 11 | 0.34 |
| MGST1 | KB742887.1 | 2540000 | 2580000 | 76 | 0.56 | 77 | 0.47 | 1 | 0.07 |
| SLC15A5 | KB742887.1 | 2600000 | 2640000 | 77 | 0.52 | 81 | 0.43 | 1 | 0.07 |
| LMO3 | KB742887.1 | 2500000 | 2540000 | 72 | 0.52 | 72 | 0.44 | 0 | 0.00 |
| SLC22A1 | KB742815.1 | 860000 | 900000 | 67 | 0.45 | 93 | 0.40 | 63 | 0.17 |
| DERA | KB742887.1 | 2680000 | 2720000 | 28 | 0.43 | 27 | 0.37 | 11 | 0.27 |
|  | KB744788.1 | 40000 | 80000 | 152 | 0.41 | 61 | 0.20 | 144 | 0.37 |
|  | KB742944.1 | 100000 | 140000 | 406 | 0.40 | 369 | 0.32 | 374 | 0.37 |
|  | KB742944.1 | 100000 | 140000 | 406 | 0.40 | 369 | 0.32 | 374 | 0.37 |
|  | KB742944.1 | 120000 | 160000 | 415 | 0.40 | 423 | 0.32 | 409 | 0.39 |
|  | KB742944.1 | 120000 | 160000 | 415 | 0.40 | 423 | 0.32 | 409 | 0.39 |
| IGF2R | KB742815.1 | 820000 | 860000 | 129 | 0.39 | 140 | 0.45 | 32 | 0.21 |
| C7orf31 | KB743583.1 | 140000 | 180000 | 431 | 0.38 | 361 | 0.29 | 431 | 0.36 |
| BPGM | KB742449.1 | 0 | 40000 | 351 | 0.37 | 331 | 0.46 | 199 | 0.16 |
|  | KB742449.1 | 0 | 40000 | 351 | 0.37 | 331 | 0.46 | 199 | 0.16 |
|  | KB742944.1 | 80000 | 120000 | 514 | 0.37 | 437 | 0.35 | 434 | 0.32 |
| NPVF | KB743583.1 | 120000 | 160000 | 367 | 0.36 | 293 | 0.30 | 362 | 0.37 |
| SFI1 | KB744033.1 | 920000 | 960000 | 68 | 0.35 | 79 | 0.46 | 59 | 0.18 |
| DRG1 | KB744033.1 | 900000 | 940000 | 70 | 0.35 | 84 | 0.45 | 38 | 0.16 |
| EIF4ENIF1 | KB744033.1 | 900000 | 940000 | 70 | 0.35 | 84 | 0.45 | 38 | 0.16 |
