# Supplementary material for: Analysis of Anasplatyrhynchos genome resequencing data reveals genetic signatures of artificial selection
Source: PLoS One. 2019 Feb 8;14(2):e0211908. doi: 10.1371/journal.pone.0211908 (PMC6368380; doi:10.1371/journal.pone.0211908)
Supplement: S14 Table — (DOCX) [file pone.0211908.s021.docx]

**S14 Table. The annotation of SNPs by *IGF2R* harbored missense variants**

| Scaffold | Position | Reference | Alternative | Variant-type | Effect | Nucleotide | Protein |
| --- | --- | --- | --- | --- | --- | --- | --- |
| KB742815.1 | 855461 | A | G | missense_variant | MODERATE | c.241A>G | p.Ile81Val |
|  | 884812 | G | A | missense_variant | MODERATE | c.5119G>A | p.Val1707Ile |
|  | 886774 | T | C | missense_variant | MODERATE | c.5509T>C | p.Trp1837Arg |
|  | 886776 | G | A | missense_variant |  | c.5511G>A |  |
|  | 865409 | A | G | synonymous_variant | LOW | c.1713A>G | p.Ala571Ala |
|  | 868650 | T | C | synonymous_variant | LOW | c.2277T>C | p.Arg759Arg |
|  | 887986 | G | A | synonymous_variant | LOW | c.5871G>A | p.Thr1957Thr |
|  | 892672 | A | T | synonymous_variant | LOW | c.6312A>T | p.Thr2104Thr |
