# Supplementary material for: Analysis of Anasplatyrhynchos genome resequencing data reveals genetic signatures of artificial selection
Source: PLoS One. 2019 Feb 8;14(2):e0211908. doi: 10.1371/journal.pone.0211908 (PMC6368380; doi:10.1371/journal.pone.0211908)
Supplement: S15 Table — (DOCX) [file pone.0211908.s022.docx]

**S15 Table. The allele frequencies of G and A at 5119G>A mutation site in the FTPD and LTPD populations**

| Species | AA | AG | GG | A | G |
| --- | --- | --- | --- | --- | --- |
| FTPD | 12 | 7 | 3 | 0.7045 | 0.2955 |
| LTPD | 0 | 4 | 19 | 0.0870 | 0.9130 |
