# Supplementary material for: Analysis of Anasplatyrhynchos genome resequencing data reveals genetic signatures of artificial selection
Source: PLoS One. 2019 Feb 8;14(2):e0211908. doi: 10.1371/journal.pone.0211908 (PMC6368380; doi:10.1371/journal.pone.0211908)
Supplement: S16 Table — (DOCX) [file pone.0211908.s023.docx]

**S16 Table. The allele frequencies of T and C at 5509T>C mutation site in the FTPD and LTPD populations**

| Species | CC | CT | TT | C | T |
| --- | --- | --- | --- | --- | --- |
| FTPD | 12 | 8 | 2 | 0.7273 | 0.2727 |
| LTPD | 0 | 5 | 18 | 0.1087 | 0.8913 |
