# Supplementary material for: Analysis of Anasplatyrhynchos genome resequencing data reveals genetic signatures of artificial selection
Source: PLoS One. 2019 Feb 8;14(2):e0211908. doi: 10.1371/journal.pone.0211908 (PMC6368380; doi:10.1371/journal.pone.0211908)
Supplement: S17 Table — (DOCX) [file pone.0211908.s024.docx]

**S17 Table. Primers used for validation the SNPs in the duck *IGF2R* gene**

| Primer name | Primer sequence(5’-3’) | SNP mutations | Tm (℃) | Product length（bp） |
| --- | --- | --- | --- | --- |
| P1 | 5' CACCAGCACTACTCCTTGTC 3'  5' CTTGTTACTCCCCACTTCTC 3' | 5119G>A | 53 | 196 |
| P2 | 5' GTGGTGTGTCATCCTTTGG 3'  5' CTTCCCTCCCTTAGCAAC 3' | 5509T>C 5511G>A | 53 | 240 |
| *ACTB* | 5' TACAGGAAGTTACTCGCC 3'  5' CATCTATCACTGGGGAAC 3' |  | 48 | 205 |
