# Supplementary material for: Analysis of Anasplatyrhynchos genome resequencing data reveals genetic signatures of artificial selection
Source: PLoS One. 2019 Feb 8;14(2):e0211908. doi: 10.1371/journal.pone.0211908 (PMC6368380; doi:10.1371/journal.pone.0211908)
Supplement: S18 Table — (DOCX) [file pone.0211908.s025.docx]

**S18 Table. Primers used for analyzing mRNA expression levels of *IGF2R* and *HCN1***

| Primer name | Primer sequence(5’-3’) | Tm (℃) | Product length（bp） |
| --- | --- | --- | --- |
| *IGF2R* | 5' GCTTCTGGTGACATACGGAC 3'  5' CCAAGTCATCATCCTCAACG 3' | 56 | 187 |
| *HCN1* | 5' ATCCTTTCGCCCTGTTCC 3'  5' CCTGGGTGCTCTTGTGAAC 3' | 56.6 | 187 |
| *ACTB* | 5' TACAGGAAGTTACTCGCC 3'  5' CATCTATCACTGGGGAAC 3' | 48 | 205 |
